# P38α MAPK Coordinates Mitochondrial Adaptation to Caloric Surplus in Skeletal Muscle

**DOI:** 10.3390/ijms25147789

**Published:** 2024-07-16

**Authors:** Liron Waingerten-Kedem, Sharon Aviram, Achinoam Blau, Tony Hayek, Eyal Bengal

**Affiliations:** 1 Department of Biochemistry, The Ruth and Bruce Rappaport Faculty of Medicine, Technion-Israel Institute of Technology, P.O. Box 9649, Bat Galim, Haifa 31096, Israel; w.liron@campus.technion.ac.il (L.W.-K.); avirams@technion.ac.il (S.A.); achinoam.bl@campus.technion.ac.il (A.B.); t_hayek@rambam.health.gov.il (T.H.); 2 Department of Internal Medicine E, Rambam Health Care Campus, P.O. Box 9602, Bat Galim, Haifa 31096, Israel

**Keywords:** skeletal muscle, high-fat diet, mitochondrial metabolism, p38α MAPK, insulin resistance, metabolomics

## Abstract

Excessive calorie intake leads to mitochondrial overload and triggers metabolic inflexibility and insulin resistance. In this study, we examined how attenuated p38α activity affects glucose and fat metabolism in the skeletal muscles of mice on a high-fat diet (HFD). Mice exhibiting diminished p38α activity (referred to as p38α^AF^) gained more weight and displayed elevated serum insulin levels, as well as a compromised response in the insulin tolerance test, compared to the control mice. Additionally, their skeletal muscle tissue manifested impaired insulin signaling, leading to resistance in insulin-mediated glucose uptake. Examination of muscle metabolites in p38α^AF^ mice revealed lower levels of glycolytic intermediates and decreased levels of acyl-carnitine metabolites, suggesting reduced glycolysis and β-oxidation compared to the controls. Additionally, muscles of p38α^AF^ mice exhibited severe abnormalities in their mitochondria. Analysis of myotubes derived from p38α^AF^ mice revealed reduced mitochondrial respiratory capacity relative to the myotubes of the control mice. Furthermore, these myotubes showed decreased expression of Acetyl CoA Carboxylase 2 (ACC2), leading to increased fatty acid oxidation and diminished inhibitory phosphorylation of pyruvate dehydrogenase (PDH), which resulted in elevated mitochondrial pyruvate oxidation. The expected consequence of reduced mitochondrial respiratory function and uncontrolled nutrient oxidation observed in p38α^AF^ myotubes mitochondrial overload and metabolic inflexibility. This scenario explains the increased likelihood of insulin resistance development in the muscles of p38α^AF^ mice compared to the control mice on a high-fat diet. In summary, within skeletal muscles, p38α assumes a crucial role in orchestrating the mitochondrial adaptation to caloric surplus by promoting mitochondrial biogenesis and regulating the selective oxidation of nutrients, thereby preventing mitochondrial overload, metabolic inflexibility, and insulin resistance.

## 1. Introduction

Obesity stands as a primary instigator of insulin resistance in peripheral tissues and the onset of type 2 diabetes (T2D) [1]. Among the various tissues, skeletal muscle takes center stage as a prime hub for glucose disposal, and as a major reservoir for glycogen storage. Thus, this tissue is a critical player in maintaining glycemic control and the overall metabolic equilibrium. Extensive research spanning decades has illuminated the connection between the intramuscular buildup of fat-derived metabolites and the progressive decline in skeletal muscle metabolism, which ultimately culminates in insulin resistance [2,3,4].

Over the years, two prominent hypotheses have ventured to explain the role of lipids in the development of insulin resistance and glucose intolerance. An earlier model, offered by Randle and colleagues, introduced the concept of the “glucose-fatty acid cycle” [5]. This model posited that as the supply of fatty acids increases, their oxidation within the mitochondria reaches saturation, consequently leading to the accumulation of acetyl CoA, NADH, and ATP. Their accumulation inhibits pyruvate dehydrogenase (PDH)—the mitochondrial enzyme that links glycolysis to glucose oxidation. Consequently, the utilization of carbohydrates is impeded, fostering the development of insulin resistance. Recent results of metabolomics studies have reinforced this model by revealing a connection between insulin resistance and the burdening of mitochondria with lipids. Under such conditions, the rates of incomplete fat oxidation are heightened and the long- and medium-chain acylcarnitines accumulate, contributing to what is termed as “metabolic inflexibility” [6,7,8]. This term denotes the inability of insulin-resistant individuals to adapt mitochondrial fuel selection in response to shifting nutritional cues, such as the switching of fat to carbohydrates during the transition from fasting to feeding. A second more recent model proposed that insulin resistance is triggered by the accumulation of intramyocellular lipids resulting from excessive fat uptake and diminished mitochondrial activity [9,10]. According to this model, lipid signaling molecules like diacylglycerols (DAGs) and ceramides, which are synthesized in the cytosol from accumulated fatty acids, activate specific stress kinases, notably PKCθ, which inhibits insulin-signaling molecules such as insulin receptor substrate (IRS) and AKT [11,12,13]. Since insulin signaling plays a pivotal role in orchestrating the trafficking of glucose transporters (GLUTs) to the plasma membrane, and in facilitating glucose uptake into cells, its inhibition disrupts the initial phase of glucose transport. Importantly, mitochondrial dysfunction is a common denominator in both models of insulin resistance.

The p38 mitogen-activated kinase (MAPK) pathway is activated by various stress stimuli, and it takes part in a multitude of cellular processes [14]. Despite its importance, our comprehension of the roles of p38 MAPK in skeletal muscle metabolism remains somewhat limited [15]. Intriguingly, the impact of p38 MAPK on metabolism hinges on the specific type of stress experienced by skeletal muscle. The activation of p38 MAPK by muscle contraction induces the adaptation of muscle fibers to increased energy requirements by enhancing oxidative metabolism and glucose uptake. Mechanistically, p38 MAPK achieves these effects by upregulating the expression of glucose transporters and the transcriptional co-activator PGC1α, thereby promoting mitochondrial biogenesis and oxidative metabolism [16,17]. Consequently, p38 MAPK largely enhances muscle oxidative capacity in an insulin-independent fashion.

The role of chronic activation of p38 MAPK in insulin resistance, which develops under conditions of caloric surplus, is debatable. While some studies have linked p38 kinase activity to the hindrance of insulin signaling by affecting IRS1 and Akt protein kinase [18,19,20,21], others have suggested its potential in preventing insulin resistance [22,23,24]. For instance, experiments in which MAPK phosphatase-1 (MKP-1) expression was altered revealed complex consequences; increased MKP-1 expression led to obesity and insulin resistance by the deactivation of p38/JNK MAPKs, whereas the deletion of MKP-1 prevented insulin resistance. Despite these findings, the precise role of p38 MAPK in insulin resistance and its impact on mitochondrial metabolism in the regulation of fat and glucose metabolism remains unexplored.

In this study, we investigated the role of p38α MAPK in skeletal muscle metabolism under a high-fat diet (HFD). We found that mice with attenuated p38α activity (p38α^AF^) were more resistant to insulin than control mice. The skeletal muscles of p38α^AF^ mice exhibited reduced mitochondrial oxidative activity relative to the muscles of control mice. Additionally, we report that p38α is indispensable for the expression of Acetyl CoA Carboxylase 2 (ACC2), which is a critical regulator of long-chain fatty acid (LCFA) oxidation, and for the regulation of pyruvate dehydrogenase (PDH), which controls pyruvate oxidation within the mitochondria. In summary, our findings emphasize the pivotal role of p38α in coordinating mitochondrial adjustments to fulfill metabolic requirements, thereby preserving insulin sensitivity in skeletal muscles.

## 2. Results

### 2.1. Mice with Attenuated p38α Activity Develop Metabolic Syndrome and Exhibit Decreased Insulin Sensitivity Relative to Wild-Type Mice

To investigate the role of p38α in metabolism under different dietary conditions (normal chow diet (ND) or a high-fat diet (HFD)), we used a mouse strain known as p38α^AF^. These mice carry two mutations in the activation loop of p38α, rendering them resistant to phosphorylation by upstream kinases. These mutations result in the attenuation of p38α MAPK signaling [25].

**Figure 1:**
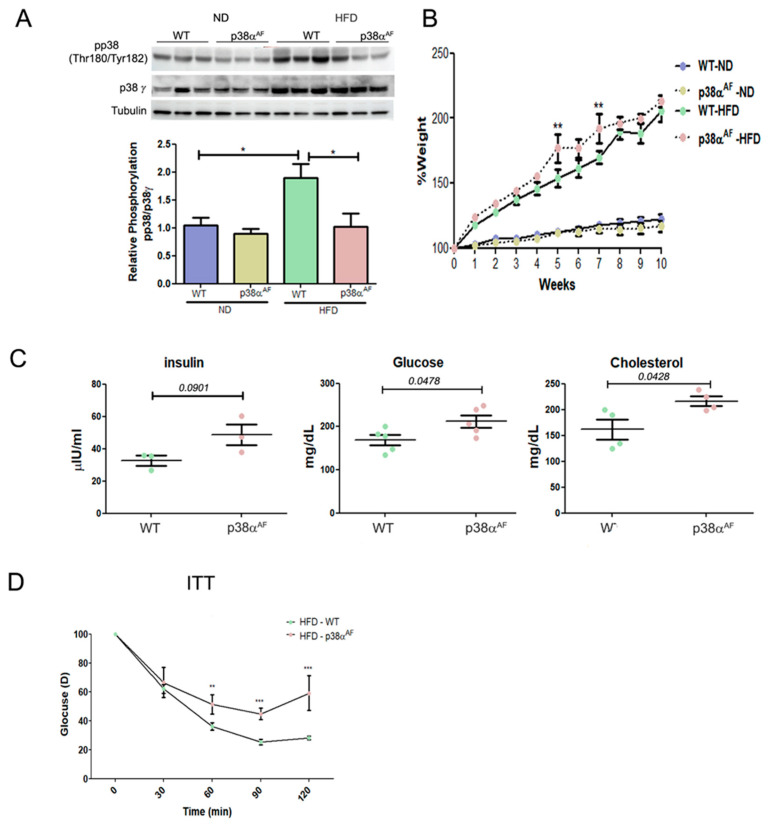
p38α^AF^ mice presented worse metabolic parameters than control mice. (**A**) Six-week-old mice were fed with ND or an HFD for 10 weeks, and GC muscles were isolated (*n* = 5) from the control and p38α^AF^ mice. Protein lysates from three of the mice per treatment were randomly analyzed by Western blotting with the designated antibodies. α Tubulin was used as the loading control. The quantification of relative p38α phosphorylation is presented in the histogram. (**B**) The mice underwent the diets described in (A), and the weight of each mouse was measured weekly (*n* = 5). The graphs represent the average percent change in the body weight of the two mouse groups (control and p38α^AF^), which were fed with ND or HFD. The weight was set to 100 on the first day of the diet. (**C**) The hematological parameters of control mice and p38α^AF^ on an HFD. The glucose and cholesterol levels were measured in the serum of control and p38α^AF^ mice after 10 weeks on an HFD (AML-central lab services). Insulin was measured (*n* = 3) using an ELISA kit (Millipore RAB0817). The significance probabilities between treatments were designated as numbers. (**D**) Insulin tolerance test (ITT): the graph displays the relative average glucose levels at 0, 30, 45, 60, 90, and 120 min following insulin injection (0.5 U/kg BW) in the blood of control and p38α^AF^ mice after a 10-week HFD (*n* = 4 mice per group). The mice were deprived of chaw for 6 h before insulin was IP-injected. The glucose level before insulin injection was set to 100 percent, and all values were relative to 100. Data are presented as the mean ± SE. One-way ANOVA was followed by Tukey post-tests (**A**), two-way ANOVA was followed by Bonferroni post-tests (**B**,**D**) and a Student t-test (**C**). The *p* values for group difference are designated as follows: * *p* < 0.05, ** *p* < 0.01, and *** *p* < 0.001.

Analysis of p38 MAPK after 10 weeks of each dietary regime revealed higher phosphorylation levels in the skeletal muscles of HFD-fed control mice compared to those fed by ND (Figure 1A). By contrast, p38α^AF^ mice displayed lower phosphorylation levels in their muscles under both diet regimens, thus confirming that p38 MAPK phosphorylation was indeed attenuated in the muscles of those mice, regardless of the diet.

While on an ND, both mouse strains experienced a similar weight gain. However, on an HDF, p38α^AF^ mice displayed a slightly higher increase in weight gain over time compared to the control mice (Figure 1B and Supplementary Materials, Figure S1). Furthermore, in the HFD group, p38α^AF^ mice exhibited increased levels of blood glucose, insulin, and cholesterol (Figure 1C). Insulin tolerance tests (ITTs) on the HFD-fed mice demonstrated that the insulin-injected p38α^AF^ mice displayed a reduced capacity to effectively remove glucose from the bloodstream compared to control mice (Figure 1D). In conclusion, the whole-body parameters indicated a disruption in the metabolic balance of p38α^AF^ mice compared to control mice.

### 2.2. The Muscles of p38α^AF^ Mice Display Compromised Insulin Signaling and Resistance to Insulin-Mediated Glucose Uptake

**Figure 2:**
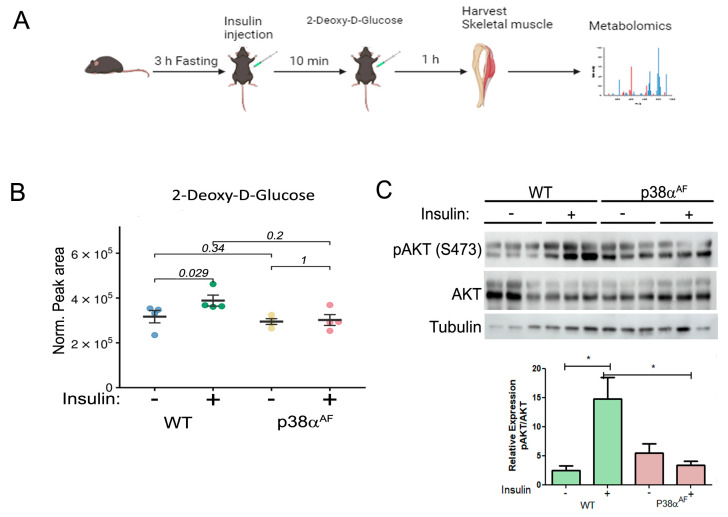
Block of the insulin-mediated 2 deoxy-glucose (2DG) uptake by the Tibialis muscle of p38α^AF^ mice. (**A**) Experimental layout: saline or insulin (1 unit/kg) was IP-injected following a 3 h fasting of the mice previously fed with an HFD for 10 weeks. Ten min later, 5% 2DG was IP-injected (10 μL to 1 g weight). The mice were sacrificed one hour later, and the Tibialis muscles were frozen and used in the mass spectrometry (MS) analysis of metabolites, or to extract proteins for Western blotting analysis. (**B**) Peak area were analyzed by the MS values of 2- Deoxy –D Glucose (*n* = 4) that were normalized to mg tissue. (**C**) Protein extracts from the Tb muscles (*n* = 3) were analyzed by Western blotting with antibodies directed to phosphorylated Akt (Serine 473) and Pan Akt. Quantification of the relative phosphorylation (pAkt/Akt) is presented in the histogram. Data are presented as the mean ± SE. The Wilcoxon test and significance probabilities between treatments are designated as numbers in (**B**). One-way ANOVA was followed by Tukey post-tests. The *p* values for group difference are designated as follows: * *p* < 0.05 (**C**).

Increased blood insulin and glucose levels could reflect insulin resistance in peripheral tissues. As skeletal muscle plays a key role in maintaining blood glucose homeostasis, we proceeded to examine the insulin-mediated glucose uptake in the muscle tissue. For that purpose, ten weeks of HFD-fed mice were administered insulin and non-metabolizable 2-deoxy-D-glucose (2DG). One hour following 2DG injection, metabolites from the Tibialis anterior (Tb) muscles were extracted and analyzed using mass spectrometry (Figure 2A).

Insulin injection elevated the 2DG levels in the muscles of control mice by approximately 20%, while it had no impact on the 2DG levels in the muscles of p38α^AF^ mice (Figure 2B). This result suggests that the insulin-mediated glucose uptake was completely inhibited in the muscles of p38α^AF^ mice, whereas it was only partially inhibited in the muscles of control mice.

Therefore, we examined insulin signaling in the muscles by analyzing Akt Serine 473 phosphorylation (Figure 2C). Insulin increased Akt phosphorylation in the muscles of control mice but not in the muscles of p38α^AF^ mice.

### 2.3. Insulin Fails to Augment Glycolysis in the Muscles of HFD-Fed p38α^AF^ Mice

**Figure 3:**
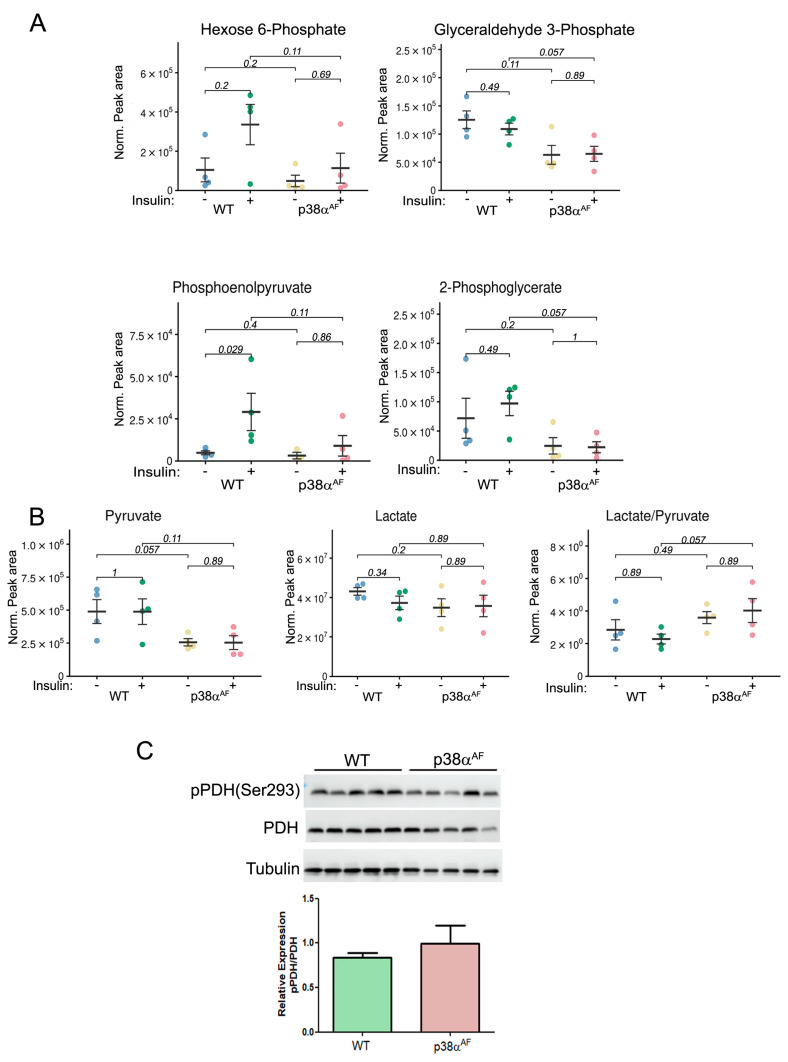
Reduced glycolytic metabolites and increased lactate-to-pyruvate ratio in the muscles of HFD-fed p38α^AF^ mice. Extracted metabolites from the Tibialis muscles of 10-week HFD-fed mice that were IP-injected without or with insulin (*n* = 4). (**A**) The normalized peak areas (to mg tissue) that were analyzed by the MS of several glycolytic metabolites. (**B**) The normalized peak areas (to mg tissue) that were analyzed by the MS of pyruvate, lactate, and the ratio of lactate to pyruvate. (**C**) Analysis of the expression and the phosphorylation on serine 293 of the E1 subunit of pyruvate dehydrogenase (PDH) in the Tb muscles of control and p38α^AF^ mice (*n* = 5) by Western blotting using antibodies to phospho-PDH (Ser293) and PDH. The quantification of relative phosphorylation (pPDH/PDH) is presented in the histogram. Data are presented as the mean ± SE. The Wilcoxon test and significance probabilities between treatments are designated as numbers in (**B**).

To assess whether insulin affects glycolytic and tricarboxylic acid (TCA) metabolites in the muscles of high-fat-diet-fed mice, we examined the metabolites in the Tibialis anterior (Tb) muscles using mass spectrometry. The levels of several glycolytic metabolites like hexose 6-phosphate (H6P: G6P and F6P), phosphoenolpyruvate, and 2-phosphoglycerate were increased to different levels by insulin treatment in the control muscles without being changed in the muscles of the p38α^AF^ mice (Figure 3A) The levels of glyceraldehyde 3-phosphate were not increased by insulin but remained lower in the muscles of p38α^AF^ mice relative to the levels in the muscles of control mice. These findings suggest that insulin increased glycolysis in the muscles of control mice but did not elicit a similar response in the muscles of p38α^AF^ mice.

Steady-state levels of pyruvate were lower in the p38α^AF^ muscles than in the muscles of control mice, whereas lactate levels were similar in the muscles of both strains (Figure 3B). Consequently, a higher lactate-to-pyruvate ratio in the p38α^AF^ muscles suggests an increased conversion of pyruvate to lactate by lactate dehydrogenase (LDH). Next, we analyzed the inhibitory phosphorylation (Ser293 of E1 subunit) of pyruvate dehydrogenase (PDH) [26]. We observed a non-significant elevation in inhibitory phosphorylation in the muscles of p38α^AF^ mice compared to those of the control mice (Figure 3C). The similar levels of TCA metabolites in the muscles of both mouse strains indicate a comparable flow through the TCA cycle (Supplementary Materials, Figure S2). Insulin treatment significantly decreased the levels of several TCA metabolites (citrate, cis-aconitate, and α-ketoglutarate) in the muscles of control mice, but this was less so in the muscles of p38α^AF^ mice. This decrease may point at specific TCA cycle intermediates that participate in the biosynthesis of amino acids [27]. Indeed, the concentrations of several amino acids were elevated in the muscles of control mice as opposed to the levels in the muscles of p38α^AF^ mice (Supplementary Materials, Figure S3). Collectively, the above findings underscore that, unlike the insulin stimulatory effect observed in the muscles of the control mice, there was a failure to augment the glycolytic and TCA cycle-derived metabolites in the muscles of the p38α^AF^ mice.

### 2.4. Reduced Fatty Acid Oxidation in the Muscles of p38α^AF^ Mice

Next, we analyzed fat metabolites that are associated with insulin resistance [28]. We detected slightly elevated levels of long-chain saturated (palmitic acid) and unsaturated FAs (arachidonic and oleic acids), but not medium-chain FAs (octanoic acid), in the muscles of p38α^AF^ mice relative to the control mice (Supplementary Materials, Figure S4).

**Figure 4:**
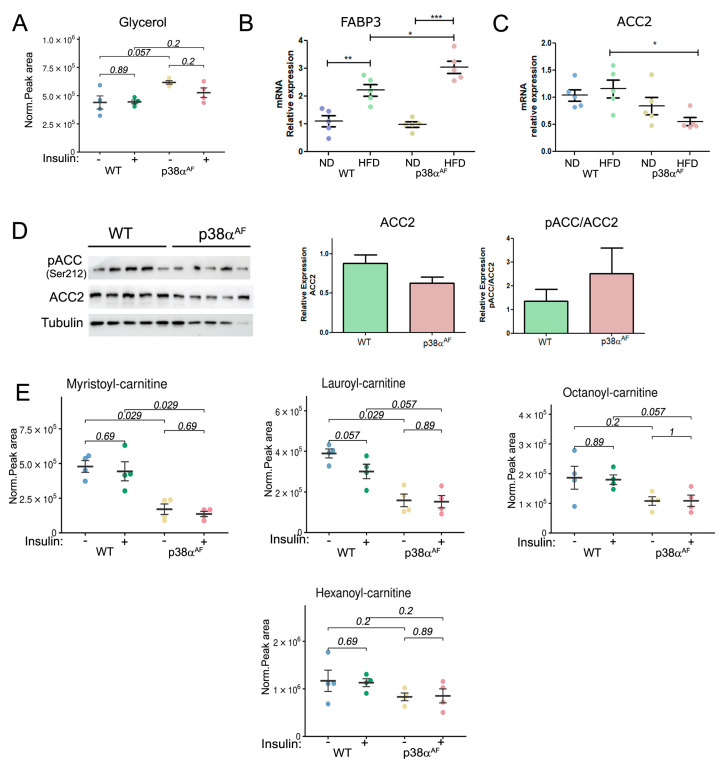
Reduced β oxidation in the muscles of p38α^AF^ mice relative to the muscles of control mice following a high-fat diet. Metabolites were extracted from the Tibialis muscles of 10-week HFD-fed mice that were IP-injected without or with insulin (*n* = 4). (**A**) The peak areas (normalized to mg tissue) of glycerol analyzed by MS are presented. (**B**) Analysis of the mRNA levels of FABP3 in the muscles of control and p38α^AF^ mice by qPCR (*n* = 5). The β-actin housekeeping gene was used to normalize the mRNA levels. (**C**) Analysis of the mRNA levels of ACC2 in the muscles of control and p38α^AF^ mice by qPCR (*n*= 4). The β-actin housekeeping gene was used to normalize mRNA levels. (**D**) Analysis of the expression and the phosphorylation on serine 212 of Acetyl CoA Carboxylase 2 (ACC2) in the muscles of control and p38α^AF^ mice (*n* = 5) by Western blotting using antibodies to phospho-ACC2 (Ser212), ACC2, and αTubulin (which served as a loading control). The histograms present the relative expression of ACC2 (ACC2/Tubulin) and relative ACC2 phosphorylation on serine 212 (pACC2/ACC2). (**E**) The peak areas (normalized to mg tissue) of acyl-carnitines are presented. Values represent the means ± SEM. The Wilcoxon test and significance probabilities between treatments are designated as numbers (**A**,**E**). One-way ANOVA followed by Tukey post-tests (**B**,**C**). The *p* values for group difference are designated as follows: * *p* < 0.05, ** *p* < 0.01, and *** *p* < 0.001.

The levels of glycerol, which is a product of triacylglycerol hydrolysis, were also higher in the muscles of p38α^AF^ mice compared to the muscles of control mice (Figure 4A). The transcript levels of Fatty Acid Binding Protein 3 (FABP3), a lipid “chaperone” [29], were increased in the muscles of the HFD-fed mice relative to its levels in the muscles of ND-fed mice. The increase in FABP3 expression in high-fat diet (HFD) conditions was particularly notable in the muscles of p38α^AF^ mice when compared to the control group (Figure 4B). These findings indicate elevated levels of stored triglycerides in the muscles of the HFD-fed p38α^AF^ mice.

The transcript levels of three proteins involved in the carnitine shuttle, were found to be similar in the muscles of both strains (Supplementary Materials, Figure S5A). Likewise, the free carnitine levels, which are necessary for long-chain fatty acid (LCFA) transportation into mitochondria, were similar in the muscles of both stains (Supplementary Materials, Figure S5B). Remarkably, the transcript levels of Acetyl CoA Carboxylase 2 (ACC2), whose product Malonyl-CoA inhibits CPT1, were significantly lower in the muscles of p38α^AF^ mice compared to control mice, especially after a high-fat diet (Figure 4C) [30]. Correspondingly, the protein levels of ACC2 were lower in the muscles of p38α^AF^ mice relative to control mice (Figure 4D). This, coupled with increased AMPK-mediated phosphorylation at Ser212, implies a potential decline in ACC2 activity, consequently impairing the inhibition of CPT1 in the muscles of p38α^AF^ mice (Figure 4D). Hence, it was expected that the transport of LCFA across the inner mitochondrial membrane should not have limited their oxidation in the muscles of p38α^AF^ mice. However, despite this expectation, we observed significantly lower levels of various mitochondrial-derived medium- and short acylcarnitine intermediates in the muscles of p38α^AF^ mice compared to those in the muscles of control mice (Figure 4E). These data suggest that the mitochondrial β-oxidation of long-chain fatty acid (LCFA) was reduced in the muscles of p38α^AF^ mice in comparison to the muscles of control mice.

### 2.5. Increased Mitochondrial Damage in the Muscles of p38α^AF^ Mice

The reduced β oxidation in the muscles of p38α^AF^ mice suggests compromised mitochondrial functionality. Consequently, we proceeded to examine the mitochondrial content and morphology in the muscles of both mouse strains. To assess the mitochondrial content, we conducted succinate dehydrogenase (SDH) activity staining on muscle sections, and we categorized them into the following three populations based on staining intensity: lightly stained (glycolytic fibers), intermediately stained (fast oxidative fibers), and intensely stained (slow oxidative fibers). The relative proportions of these three fiber types are comparable between the Tibialis muscles of control and p38α^AF^ mice under both types of diets (Supplementary Materials, Figure S6). Intriguingly, the dietary composition did influence the relative distribution of the fiber types; the percentage of intensely stained fibers decreased, while that of intermediately stained fibers was proportionally increased in the Tibialis muscles of the HFD-fed mice compared to the ND mice. However, diminished p38α activity in the muscles of p38α^AF^ mice did not affect mitochondrial content.

**Figure 5:**
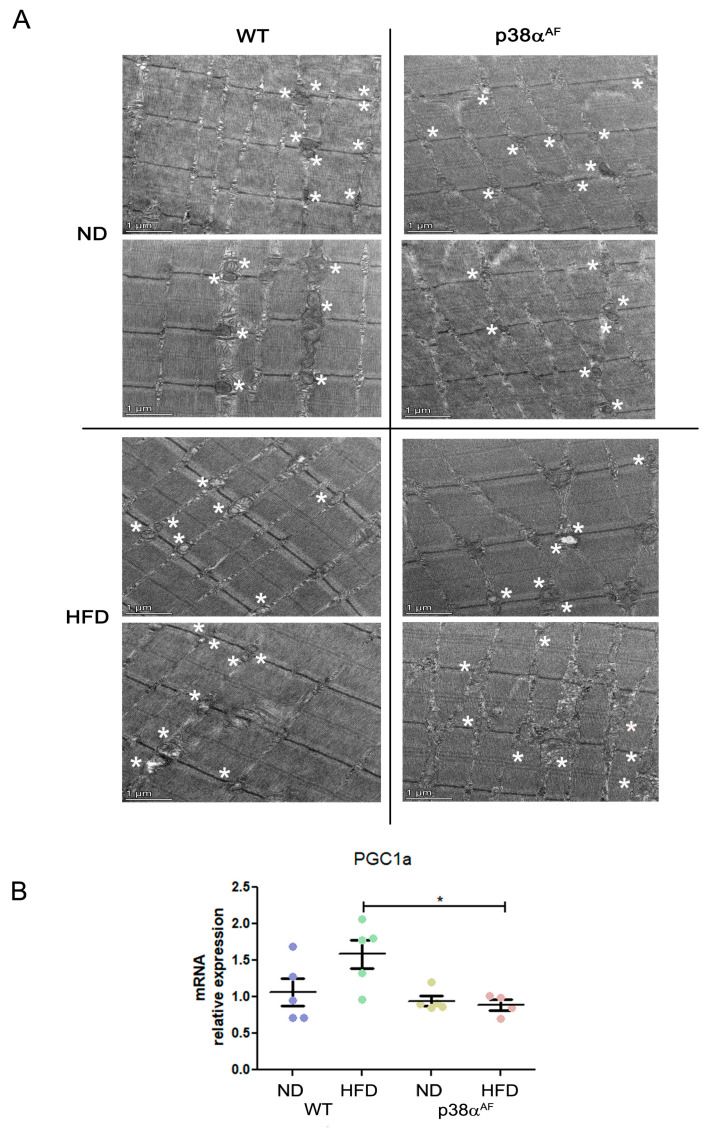
Severe mitochondrial defects in the muscles of p38α^AF^ mice. (**A**) Transmission electron microscopy (TEM) analysis of the representative muscles from control and p38α^AF^ mice fed with NDs and HFDs. The Tibialis muscles were isolated, and longitudinal sections were processed for TEM analysis (see Section 4.10). Representative images are shown. Scale bar: 1 μm. Asterisks are adjacent to the mitochondria (**B**) Analysis of the mRNA levels of PGC1α in the muscles of control and p38α^AF^ mice fed with NDs and HFDs by qPCR (*n* = 5). The β-actin housekeeping gene was used to normalize the mRNA levels. Data represent the means ± SEM. One-way ANOVA was followed by Tukey post-tests (B). The *p* values for group differences are designated as follows: * *p* < 0.05.

To gain insight into the mitochondrial quality, we analyzed the Tibialis muscles by electron microscopy. There were noticeable diet-affected differences in the mitochondrial morphology between the strains. Under a regular chow diet, the mitochondria in control muscles appeared uniform and rich in cristae, while the mitochondria of the p38α^AF^ muscle were smaller, more variable, and deformed, as well as occasionally appeared empty, punctured, or contained fat (Figure 5A). On a high-fat diet, the muscles of the control mice exhibited a decrease in mitochondria size, and this was sometimes accompanied by diminished cristae and accumulation of fat. Most of the identified mitochondria of the p38α^AF^ mice-derived muscles displayed severe structure deformities, including fat accumulation, occasional vacuolization, and membrane ruptures. The considerable morphological differences observed suggest that the mitochondria in the muscles of p38α^AF^ mice were significantly damaged compared to those of control mice, even those under a regular chow diet. Given the role of p38α in stimulating PGC1α, and thus in mitochondrial biogenesis, we examined *pgc1α* expression in the muscles of both mouse strains under different diets (Figure 5B). Notably, while the control mice showed increased *pgc1α* expression in response to a high-fat diet, its levels remained unchanged in the p38α^AF^ mice. Hence, it can be inferred that the absence of p38α activity may hinder mitochondrial biogenesis (which is particularly evident in mice on a high-fat diet), leading to the accumulation of damage.

### 2.6. Palmitate Inhibits Glycolysis, Particularly in Myotubes Derived from p38α^AF^ Mice

**Figure 6:**
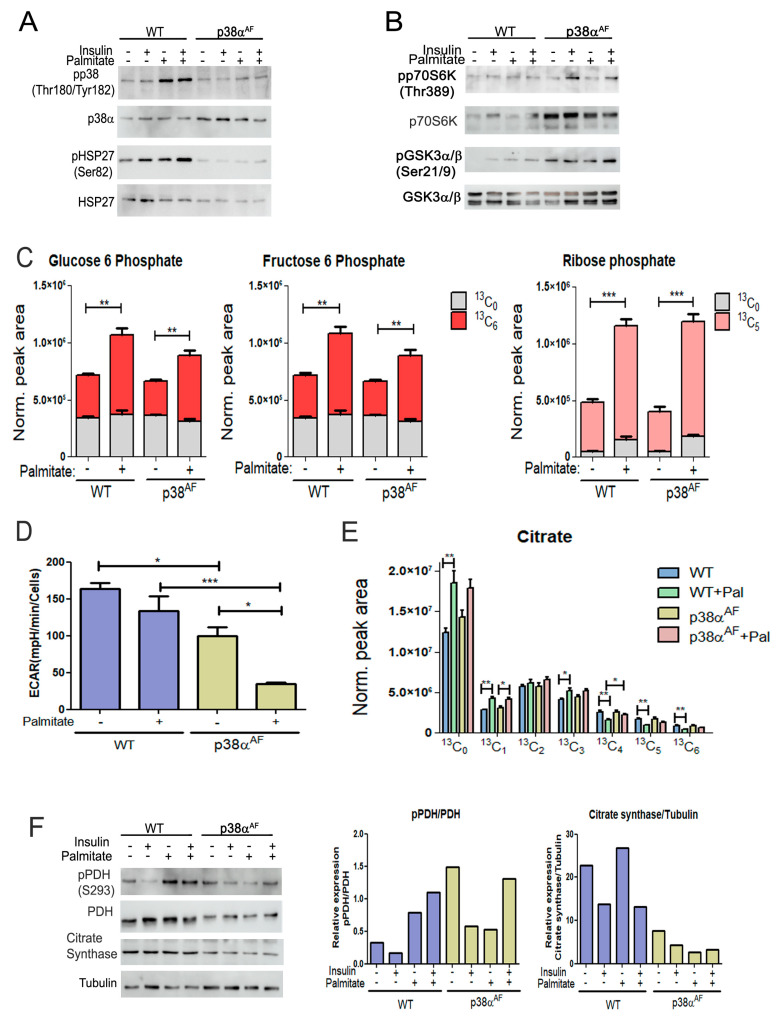
Biochemical and metabolic analysis of the myotubes derived from control and p38α^AF^ mice. (**A**) p38 MAPK phosphorylation: Myotubes were grown for 24 h in the absence or presence of 0.4 mM of palmitate. Insulin (10 μg/mL) was added 30 min before the proteins were extracted and analyzed by Western blotting using the designated antibodies. (**B**) Insulin signaling pathway: The same protein samples as in (A) were analyzed by Western blotting using the designated antibodies. (**C**) Metabolism of the (U-^13^C_6_) glucose in myotubes: (U-^13^C_6_) glucose was introduced to the myotube media with or without 0.4 mM of palmitate for 24 h. The relative levels of glucose 6-phosphate (+6), fructose 6-phosphate (+6), and ribose phosphate (+5) isotopologues are presented. The peak area was normalized to protein concentration. (**D**) Medium acidification (ECAR) of myotubes in a “Seahorse” analysis: Myotubes were grown in glucose, or glucose and palmitate, for 24 h before analysis. (**E**) Metabolism of the (U-^13^C_6_) glucose in myotubes: The relative levels of the isotopologues of citrate are presented. The peak areas were normalized to protein concentration. (**F**) Mitochondrial enzymes: The same protein samples as in (A) were analyzed by Western blotting. The histograms present the relative expression and phosphorylation of PDH (Ser293), and the expression of citrate synthase. Data represent the means ± SEM. The Wilcoxon test and significance probabilities between treatments are designated as follows: * *p* < 0.05, ** *p* < 0.01, and *** *p* < 0.001 (**C**,**E**). One-way ANOVA was followed by Tukey post-tests (**D**). The *p* values for group differences are designated as follows: * *p* < 0.05 and *** *p* < 0.001.

To elucidate whether p38α regulates the mitochondrial oxidation of glucose and fatty acids, we conducted a thorough metabolic analysis in myotubes derived from both mouse strains. The myotubes were grown on glucose alone or in combination with palmitate. In the presence of palmitate, the control myotubes exhibited an increase in the phosphorylation of p38α (Thr180/Tyr182) and its substrate, HSP27 (Ser82), while, as expected, the p38α^AF^ myotubes did not show this increase (Figure 6A). Insulin mildly increased the phosphorylation of substrates, namely p70S6K (Thr389) and GSK3 (Ser9), with this increase remaining unaffected by the inclusion of palmitate in the growth media (Figure 6B).

To explore the impact of palmitate on glucose oxidation, we incorporated heavy-carbon (U-^13^C_6_) glucose into the growth media, both with and without palmitate, for a duration of 24 h. Subsequently, we examined the metabolic pattern of glucose-derived isotopologues using mass spectrometry. Initially, we focused on glycolytic metabolites. The levels of glucose-6-phosphate (G6P)(+6) and fructose 6-phosphate (F6P)(+6) were comparable in both types of myotubes, and the addition of palmitate to the growth medium caused a significant increase in their levels. The accumulation of both metabolites suggests that palmitate inhibited phosphofructokinase (PFK) activity (Figure 6C).

The presence of palmitate also increased ribose phosphate (+5), indicating a potential shunting of G6P (+6) toward the pentose phosphate and nucleotide synthesis pathways (Figure 6C). The findings suggest that the oxidation of palmitate led to a reduction in glycolysis. To directly investigate the impact of palmitate on glycolysis, we evaluated myotube medium acidification through “Seahorse” analysis (Figure 6D). The results revealed a decreased glycolytic rate in the p38α^AF^ myotubes compared to the control myotubes. Furthermore, medium acidification was further reduced in the presence of palmitate, particularly in the p38α^AF^ myotubes. We concluded that homolactic fermentation in the myotubes from p38α^AF^ mice was lower than in those from control mice, especially when palmitate was included in the growth medium.

### 2.7. Reduced Regulation of Pyruvate Dehydrogenase in the Myotubes of p38α^AF^ Mice

Next, we examined the impact of palmitate oxidation on the metabolism of pyruvate (+3) in the tricarboxylic acid (TCA) cycle. An analysis of the isotopologues of citrate, which is the product of citrate synthase, revealed a comparable incorporation of pyruvate (+3) into the TCA cycle of both types of myotubes that were grown on glucose (+6) (Figure 6E). Adding unlabeled palmitate to the growth medium increased the levels of unlabeled citrate (+0, +1) while it reduced the levels of heavily labeled citrate (+4, +5, +6) to a lesser extent in the p38α^AF^ myotubes compared to the control myotubes.

Interestingly, unlabeled palmitate increased the levels of citrate (+3), indicating a heightened anaplerotic activity of pyruvate carboxylase (PC). Together, these observations suggest that oxidation of palmitate in the TCA cycle decreases pyruvate incorporation through pyruvate dehydrogenase (PDH) while simultaneously increasing its incorporation via PC in the control myotubes, and this also applies to a lesser extent in the p38α^AF^ myotubes.

To understand this phenomenon, we analyzed the inhibitory phosphorylation of the pyruvate dehydrogenase (PDH) complex, which regulates the flow of carbohydrates for oxidation in the TCA cycle [31]. Palmitate increased inhibitory phosphorylation on Ser293 of PDH in the control myotubes but not in the p38α^AF^ myotubes (Figure 6F). Interestingly, the expression levels of pyruvate dehydrogenase (PDH, E1 subunit) and citrate synthase (CS) were lower in the myotubes derived from the p38α^AF^ mice compared to those from the control mice. Therefore, p38α plays a vital role in regulating the expression of mitochondrial enzymes, and it participates in facilitating the inhibitory phosphorylation of PDH that is caused by palmitate. This involvement suggests a regulatory role of p38α in controlling the pyruvate decarboxylation within the mitochondria.

### 2.8. Elevated Flux of Palmitate Oxidation in the Myotubes of p38α^AF^ Mice

**Figure 7:**
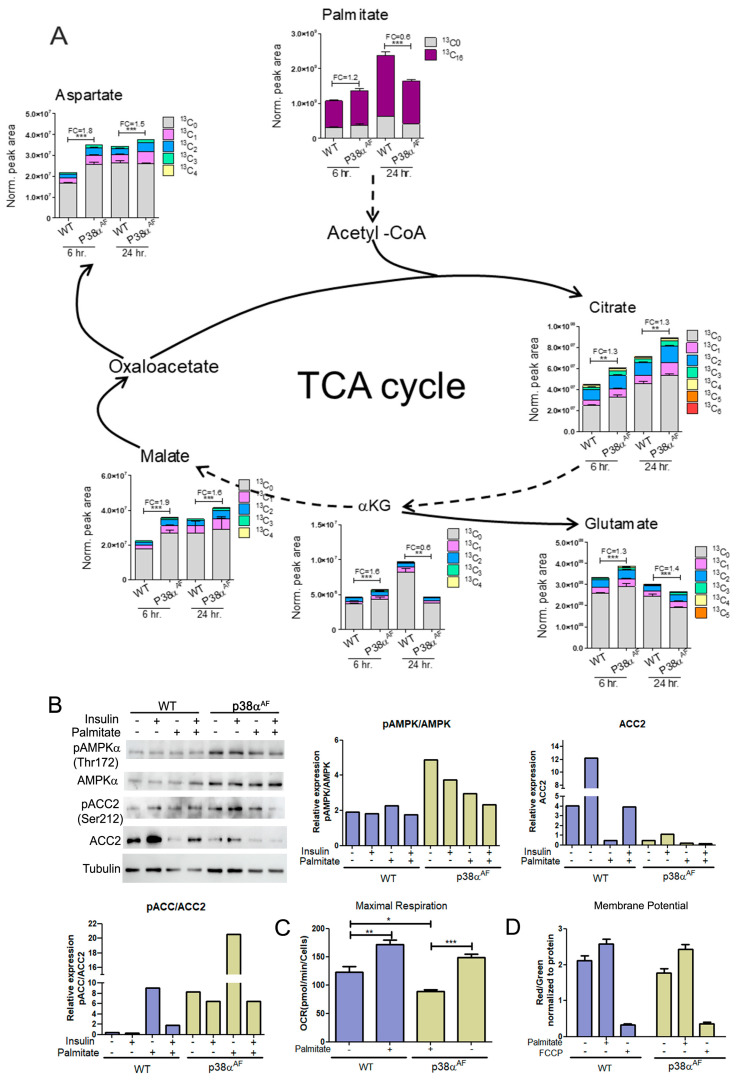
Metabolism of palmitate in the myotubes derived from control and p38α^AF^ mice. Myotubes were grown in a low-glucose DMEM supplemented with 0.4 mM of palmitate-^13^C_16_ for 6 and 24 h. (**A**) The peak area (normalized to protein concentration) of palmitate (+16), the isotopologues of the TCA cycle, and the derived amino acids that originated from palmitate-^13^C_16._ FC: fold change in the palmitate derived (^13^C ≥ 2) metabolite abundance relative to a WT of 6 h or WT of 24 h. Dashed arrows indicate of missing stages in the TCA-cycle. (**B**) Myotubes were grown for 24 h in the absence or presence of 0.4 mM of palmitate. Insulin (10 μg/mL) was added 30 min before proteins were extracted and analyzed by Western blotting with the designated antibodies. The histograms present the relative expression of ACC2, the phosphorylation of ACC2 (Ser212), and the phosphorylation of AMPKα (Thr172). (**C**) The oxygen consumption rate (OCR) at the maximal respiration of myotubes that were grown on glucose, or glucose and palmitate, for 24 h. (**D**) Comparison of the mitochondrial membrane electrochemical potential in myotubes that were grown on glucose, or glucose and palmitate, for 24 h. JC-1 dye was used to monitor the mitochondrial membrane potential. FCCP disrupts the mitochondrial membrane potential. Data represent the means ± SEM. One-way ANOVA was followed by Tukey post-tests (**A**,**C**). The *p* values for group difference are designated as follows: * *p* < 0.05, ** *p* < 0.01, and *** *p* < 0.001.

To investigate the mitochondrial oxidation of the palmitate in the TCA cycle, palmitate-^13^C_16_ was added into a glucose-based media (DMEM) in which myotubes were grown for 6 and 24 h (Figure 7). At 6 h, the levels of palmitate-^13^C_16_ were similar in the myotubes of both strains. However, after 24 h, the levels of palmitate-^13^C_16_ were lower in the myotubes of the p38α^AF^ mice than in the myotubes of the control mice, indicating either a reduced import or increased metabolism of the palmitate in the p38α^AF^ myotubes (Figure 7A). Next, we analyzed TCA cycle metabolites derived from palmitate-^13^C_16_. After 6 and 24 h of incubation, the isotopologues of citrate, α-ketoglutarate (and its amino acid derivative glutamate), malate, and aspartate (the derivative of oxaloacetate) were more abundant in the p38α^AF^ myotubes compared to their levels in the control myotubes (Figure 7B). The data imply that palmitate was oxidized more effectively in the mitochondria of the p38α^AF^ myotubes. To clarify whether elevated palmitate oxidation in the mitochondria of the p38α^AF^ myotubes is regulated at the crucial step of transport into the mitochondria via the carnitine shuttle, we examined the Acetyl CoA Carboxylase 2 (ACC2) enzyme, whose activity inhibits Carnitine Palmitoyltransferase 1 (CPT1). The myotubes from p38α^AF^ mice exhibited negligible ACC2 levels compared to those in the control myotubes (Figure 7B). The levels of ACC2 were further reduced in the myotubes cultured in the presence of palmitate. Moreover, AMP-activated kinase (AMPK), which is responsible for phosphorylating ACC2, thereby reducing its activity, exhibited high expression and phosphorylation (at Thr 172) in the myotubes of p38α^AF^ mice [32]. The AMPK-mediated phosphorylation of ACC2 on serine 212 was, respectively, elevated in the myotubes of p38α^AF^ mice (Figure 7B). In summary, the above results strongly suggest the unrestricted mitochondrial transport and oxidation of palmitate in the myotubes of p38α^AF^ mice. The elevated palmitate oxidation observed in the p38α^AF^ myotubes contrasted with the minimal oxidation of LCFA that was observed in the muscles of p38α^AF^ mice in vivo (see Figure 4E). This apparent paradox is discussed below (see “Section 3.5”).

### 2.9. The Myotubes from p38α^AF^ Mice Exhibited Lower Mitochondrial Capacity Compared to the Control Myotubes

To evaluate the mitochondrial respiratory capacity of myotubes and examine their responsiveness to different carbon sources (glucose or palmitate), we conducted a “Seahorse” analysis (Figure 7C). We observed that the maximal respiratory potential of p38α^AF^ myotubes grown on glucose was lower than that of control myotubes. The addition of palmitate to the growth medium increased the mitochondrial respiratory potential in both types of myotubes compared to those grown solely on glucose.

An assessment of the inner mitochondrial membrane potential using JC1 dye revealed that, irrespective of the carbon sources in the growth medium, the mitochondrial membrane potentials of control myotubes were higher than those of p38α^AF^ myotubes (Figure 7D). In summary, the described results indicate that the mitochondrial respiratory potential in the p38α^AF^ myotubes was compromised relative to the control myotubes.

## 3. Discussion

The findings of this study support the key role of p38α in regulating the insulin-mediated glucose metabolism within skeletal muscles. It is evident that p38α is indispensable for maintaining the overall health and biogenesis of the mitochondria in skeletal muscle, as well as for regulating the choice between glucose and fat oxidation within the TCA cycle. These functions of p38α are of paramount importance in preserving the insulin sensitivity of skeletal muscle.

### 3.1. P38α Mouse Model

This study utilized an in vivo mouse model that features a heterozygous mouse carrying an inactive allele of p38α (known as p38α^AF^). This model demonstrates decreased p38α activity across all tissues without indirectly influencing other MAPK family members [25]. The choice of this model over a conditional p38α knockout in skeletal muscle is justified by the significant increase in p38γ activity that is observed in the latter, which leads to muscle degeneration [33]. On the contrary, reduced p38α activity in the p38α^AF^ model lessened muscle atrophy and degeneration in advanced age [34] and denervated muscles [33]. The studies were conducted using male mice; therefore, the conclusions are limited to males. To explore potential sex differences in the activity of p38α in skeletal muscle metabolism, future studies should also include female mice.

### 3.2. Metabolomics

In this study, we utilized targeted metabolomics and metabolite profiling. The LC-MS separation method employed provides high-resolution and accurate mass measurements for numerous small metabolites. However, it should be noted that some metabolites are not effectively extracted and separated by this method. Complementary techniques, such as Gas-Chromatography (GC)-MS, could expand the range of detectable metabolites, particularly carboxylic acids and sugars.

### 3.3. P38α and Insulin Sensitivity

Our study examined skeletal muscle insulin sensitivity following a 10-week high-fat diet (HFD). The analyses showed more pronounced insulin resistance in the p38α^AF^ mice compared to the control mice. Firstly, the p38α^AF^ mice exhibited higher levels of serum glucose and insulin, and their ability to clear glucose from the blood after insulin injection (ITT) was impaired compared to the control mice. Secondly, insulin injections led to increased Akt phosphorylation (Ser 473) and elevated 2-deoxy-glucose levels in the muscles of the control mice, but not in those of the p38α^AF^ mice. Thirdly, insulin treatments raised certain glycolytic metabolite levels in the skeletal muscles of control mice, but not in those of p38α^AF^ mice. Consequently, we concluded that the skeletal muscles of p38α^AF^ mice exhibited a more pronounced insulin resistance than those of control mice.

### 3.4. P38α Regulation of β Oxidation

The findings of the present study align with past studies that support the role of p38 MAPK in preventing obesity and insulin resistance in mice on an HFD [22,24]. The metabolic analyses presented here indicate, for the first time, that p38α is crucial in maintaining insulin sensitivity by positively affecting mitochondrial integrity and balancing the oxidation of fatty acids and glucose in the mitochondria. The in vivo metabolic analysis highlighted a significantly lower fatty acid β-oxidation in the muscles of the p38α^AF^ mice compared to the muscles of the control mice. Furthermore, electron microscope images revealed an increased accumulation of fat and damage in the mitochondria of p38α^AF^ mice. The reduced β-oxidation and fat accumulation observed in the muscles of p38α^AF^ mice may arise from either an insufficient volume of functional mitochondria or the selective inhibition of long-chain fatty acid (LCFA) transport into the mitochondria. Our results indicate that the mitochondrial transport of LCFAs is not hindered and may be enhanced in the muscles of p38α^AF^ mice. First, the transcript levels of *CPT1*-, *CACT*-, and *CPT2*-encoding proteins associated with LCFA transport into the mitochondria were found to be similar in the muscles of both mouse strains. Second, the level of free carnitine, which is essential for LCFA transport into the mitochondria, was comparable between the muscles of the p38α^AF^ mice and control muscles. Third, the level of the ACC2 enzyme, which produces Malonyl-CoA, an inhibitor of CPT1 activity, was lower in the muscles of the p38α^AF^ mice compared to the level in the control muscles. Therefore, the transport of LCFA into the mitochondria appears to not be the limiting factor for β-oxidation in the muscles of p38α^AF^ mice. Instead, reduced β-oxidation may be attributed to a lower number of functional mitochondria in the muscles of the above mice. We did not find evidence of lower mitochondrial abundance in the muscles of p38α^AF^ mice. However, we did observe substantial morphological abnormalities such as small size and the presence of fatty and damaged mitochondria, particularly after a high-fat diet. Our findings suggest that reduced β-oxidation in the muscles of p38α^AF^ mice likely stems from increased mitochondrial damage and reduced functionality.

### 3.5. The Role of p38α in Mitochondrial Metabolic Flexibility

**Figure 8:**
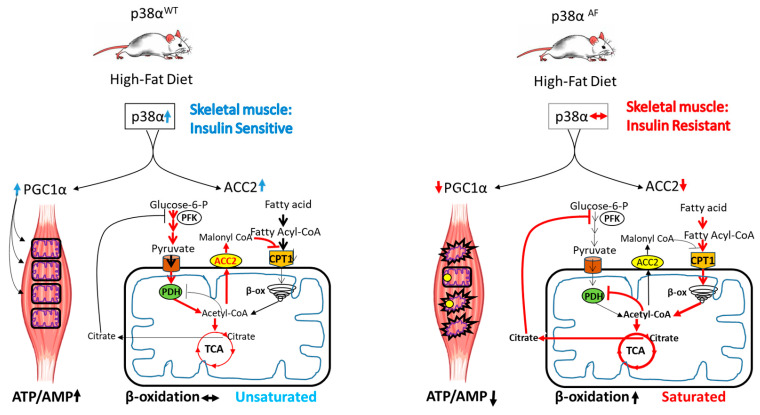
A model for the role of p38α in insulin sensitivity. In the left panel, a high-fat diet activates p38α in wild-type mice, leading to an increased expression and activity of PGC1α and ACC2 in the skeletal muscles. PGC1α acts as a co-activator, increasing mitochondrial biogenesis and activity, while ACC2 regulates fatty acid transport into mitochondria. These activities of p38α help coordinate glucose and fat oxidation, preserving metabolic flexibility and preventing mitochondrial damage. Under these conditions, both energy balance and insulin sensitivity are preserved.

To obtain a better insight into the role of p38α in insulin sensitivity, we extended the investigation to analyze the myotubes derived from the skeletal muscles of the above mouse strains. These myotubes were cultured in either glucose or palmitate, thus providing a model for the real-time assessment of glycolysis, β-oxidation, and the tricarboxylic acid (TCA) cycle. This was achieved by monitoring the substances originating from heavy-carbon-labeled (^13^C) glucose or palmitate, and these were coupled with Seahorse analysis to measure respiration. Despite the fundamental differences between this in vitro model and the in vivo model, the results provided valuable insights into the potential development of insulin resistance in the skeletal muscles of p38α^AF^ mice in vivo. Primarily, our observations showed that the myotubes derived from p38α^AF^ mice displayed reduced maximal mitochondrial respiratory potential compared to the control myotubes when cultivated with glucose or palmitate. These findings underscore the crucial role of p38α in mitochondrial biogenesis and in the mitochondrial adaptation to changing nutrients. Interestingly, our in vitro analysis indicates that the mitochondrial potential in myotubes is sufficient to adjust to their metabolic needs, unlike the evidence indicating mitochondrial deficiency and metabolic inflexibility in the skeletal muscles of p38α^AF^ mice in vivo. The muscles of p38α^AF^ mice exhibited insulin resistance on a high-fat diet (HFD), whereas the myotubes derived from these mice did not develop insulin resistance when exposed to the long-chain fatty acid (LCFA), i.e., palmitate. We propose that this variation stems from the mitochondrial adequacy in vitro versus mitochondrial inadequacy in vivo. The energy demand for the contracting muscles in vivo, which is absent in the myotubes in vitro, significantly boosts mitochondrial energy production, leading to mitochondrial stress. This stress is heightened in p38α^AF^ mice due to impaired mitochondrial biogenesis, leading to pronounced mitochondrial deficiency and damage. Besides its role in mitochondrial biogenesis, p38α appears to play a crucial role in regulating the selection of the carbon sources within the mitochondria (Figure 8). Randle’s “glucose-fatty acid cycle” theory suggests that the competition between glucose and fatty acids as mitochondrial substrates might explain the phenomenon of insulin resistance [5]. According to Randle’s theory, excessive β-oxidation of long-chain fatty acids inhibits pyruvate oxidation by the pyruvate dehydrogenase complex (PDH) in mitochondria and the glycolytic enzyme phosphofructokinase (PFK) in the cytoplasm of skeletal muscles [31]. Our findings suggest that p38α regulates the flux of β-oxidation specifically at the crucial step of long-chain fatty acid transport into the mitochondrial matrix. We identified Acetyl Co-A Carboxylase 2 (ACC2) as a significantly under-expressed enzyme in the skeletal muscles of p38α^AF^ mice compared to the muscles of control mice. ACC2 catalyzes the carboxylation of Acetyl-CoA to Malonyl-CoA, which inhibits Carnitine Palmitoyltransferase 1 (CPT1) activity, thus impeding the transport of long-chain fatty acids into the mitochondria. Earlier studies have indicated that myogenic factors, retinoic acid, and fat play roles in regulating the transcription of ACC2 in muscles [35,36,37]. The current study underscores the significance of p38α as an additional regulator of ACC2 expression as it plays a pivotal role in facilitating the transport of long-chain fatty acids (LCFA) into mitochondria.

Intriguingly, in vitro studies have suggested that p38α also plays a role in regulating the mitochondrial decarboxylation of pyruvate by PDH in myotubes. This conclusion is corroborated by the observation that, while the mitochondrial oxidation of palmitate led to a notable decrease in pyruvate oxidation in the myotubes of control mice, it had a lesser impact on pyruvate oxidation in the myotubes of p38α^AF^ mice. The absence of the inhibitory phosphorylation of PDH in response to palmitate in the myotubes of p38α^AF^ mice further supports the role of p38α in this crucial regulatory step.

Overall, a decrease in p38α activity is associated with diminished mitochondrial volume and a defective selection of carbohydrate and fatty acid oxidation, which is anticipated to overflow mitochondrial oxidation capacity, cause oxidative damage, and induce insulin resistance.

### 3.6. The Proposed Model

Based on the findings of this study, we propose a model elucidating the role of p38α in coordinating the adaptive responses of skeletal muscles to meet their energetic demands (Figure 8). P38α demonstrates a dual role: firstly, it enhances mitochondrial biogenesis by activating PGC1α; secondly, it finely regulates the flow of fatty acids and glucose toward oxidation within the mitochondria by controlling the synthesis of ACC2 and the inhibitory phosphorylation of PDH, respectively. These dual regulatory functions equip p38α with the ability to provide adaptability to mitochondria, thus enabling the selective oxidation of either fats or carbohydrates based on physiological demands. Moreover, by preventing mitochondrial saturation and reducing respiratory burden, p38α diminishes the oxidative stress caused by free radicals, thus preserving mitochondrial integrity and insulin sensitivity.

In the right panel, reduced p38α activity in the muscles of p38α^AF^ HFD-fed mice results in low PGC1α and ACC2 expression. Diminished PGC1α hinders the mitochondrial biogenesis that is associated with increased damage and decreased mitochondrial activity. Decreased levels of ACC2 allow for unregulated fatty acid transport and excessive β-oxidation, which interferes with the activity of the PDH complex and pyruvate oxidation in the TCA cycle. Consequently, impaired p38α activity results in deregulated fat and glucose oxidation, leading to metabolic inflexibility, mitochondrial overflow, and oxidative stress. These circumstances contribute to diminished energy balance and insulin resistance.

## 4. Materials and Methods

### 4.1. Animal Ethics

All experimental protocols were approved by the Institutional Committee for Animal Care and Use at the Technion, Israel Institute of Technology, Faculty of Medicine, Haifa, Israel (approval number IL-094-08-20). All study procedures complied with the guidelines of the NIH Guide for the Care and Use of Laboratory Animals.

### 4.2. Animal Model

The animal model utilized in this study comprised B6.Cg-Mapk14(WT)/p38^AF^ mice obtained from The Jackson Laboratory. This mouse strain serves as a haploinsufficient genetic model, where the substitution of Thr180 to Ala and Tyr182 to Phe diminishes p38α activity by abolishing the catalytic site phosphorylation and kinase activity in one allele. The p38α^AF^ allele encodes a dominant-negative p38α isoform, such that a mutation in one allele is adequate to selectively suppress p38α signaling in vivo [25]. The homozygous offspring for the mutation (p38α^AF^/^AF^) did not survive birth. Experiments in this study involved 6-week-old male mice, which were subjected to the specified diets for an additional 10 weeks.

### 4.3. HFD-Induced Obesity and Insulin Resistance

Six-week-old male C57BL/6 mice were fed basic chow (ND, 15% calories from fat) or a high-fat diet (HFD, 60% calories from fat, ENVIGO TD.06414) for ten weeks to develop obesity-induced insulin resistance [38].

### 4.4. Protein Extraction and Western Blot Analysis

Frozen gastrocnemius or Tibialis anterior muscles were immersed in RIPA lysis buffer (150 mM of NaCl, 5 mM of EDTA with a pH of 8.0, 50 mM of Tris with a pH of 8.0, 1%NP-40, 0.5% sodium deoxycholate, and 0.1% SDS in DDW) containing a protease inhibitor cocktail (#88666, Thermo Fisher Scientific, Waltham, MA, USA) and phosphatase inhibitor cocktail (#88667, Thermo Fisher Scientific, Waltham, MA, USA). Muscles were then homogenized using a bullet blender, incubated on ice for 30 min, and then cleared by centrifugation (13,000 rpm) for 20 min at 4 °C. The supernatant was collected, and protein concentrations were determined by a Bio-Rad protein assay (#5000006, Bio-Rad laboratories, Hercules, CA, USA). Equal amounts of extracted proteins (30 μg) were separated by SDS-PAGE, which were then transferred onto a nitrocellulose membrane and analyzed by Western blotting (as described in Odeh et al. [33]). Fisher Scientific, MA, USA The primary antibodies used are described in Fisher Scientific, MA, USA the Supplementary Materials section.

### 4.5. Quantitative Real-Time PCR (qRT-PCR)

Quantitative real-time PCR was carried out using the QuantStudio™ 3 Real-Time PCR System (Applied Biosystems, Thermo Fisher Scientific, MA, USA). Then, 18 µL of SYBR Premix Ex Taq^TM^ II reaction mix (#RR820A, TaKaRa Bio, Japan) containing the desired primers were added to 2 µL of cDNA. The PCR conditions were as follows: an initial 2 min induction step at 95 °C, followed by 40 cycles of amplification: 20 s at 95 °C, 30 s at 60 °C, and 30 s at 72 °C. The run was terminated by a melting curve from 95 °C to 60 °C to ensure PCR product purity. Two negative controls were used to verify the authenticity of the results: the reverse transcriptase negative control from the cDNA synthesis step and the water-only control. Beta-actin was used as a loading control, and all values were normalized to its levels.

### 4.6. Targeted Metabolomics and Stable Isotope Tracing Analysis by LC-MS

#### 4.6.1. Sample Preparation

Tibialis muscles: Precellys Lysing Kits comprising prefilled tubes with beads (CK28R homogenizing tubes for hard tissue containing 2.8 mm of ceramic beads) were prefilled with 500 μL of cold (−20 °C) metabolite extraction solvent (methanol, acetonitrile, and water at a 5:3:2 ratio) and kept on ice. Frozen muscle tissue was sliced, and 20–30 mg of sample was added to the homogenization tube. The samples were homogenized at approximately 4 °C using Precellys 24 homogenizer with the following parameters: 3 × 30 s, with a 30 s gap between each of the three cycles at 6500 RPM. The homogenates were centrifuged at 16,100× *g* for 10 min at 4 °C. The supernatant was collected into a microcentrifuge tube, centrifuged again, and transferred to HPLC glass vials.

Myogenic progenitor cell stable isotope tracing using ^13^C_6_-Glucose or ^13^C_16_-Palmitate process: A total of 1 × 10^6^ myogenic progenitor cells derived from the muscles of control mice or p38α^AF^ mice were seeded on a 6-well plate. Confluent cells were differentiated in 2% horse serum (HS) in DMEM for 24 h, which was then followed by 24 h in 2% HS in DMEM low glucose (1.0 g/L D-Glucose) (Biological Industries, Beit Haemek, Israel). This was then replaced by 2% HS in DMEM without glucose and supplemented with 10 mM of ^13^C_6_-Glucose. After 24 h incubation at 37 °C, metabolites were extracted from 5 replicates. One plate from each strain was treated the same, only without the ^13^C_6_-Glucose labeled media for the controls. The different treatments are described in the manuscript. The same protocol was used for the palmitate-tracing experiment, with the exception that 0.4 mM of ^13^C_16_-Palmitate- was introduced into the low glucose-based media (DMEM, 1 g/L D-Glucose). ^13^C_16_-Palmitate was conjugated to bovine serum albumin (BSA) in DMEM, and BSA in DMEM was used as control. The myotubes were grown for 6 and 24 h before the addition of an extraction buffer. After the incubation period with the tracers, the cells were quickly washed with cold PBS, and metabolites were extracted with 500 μL of cold extraction buffer (same as above). The plates were put on a shaker for 15 min at 4 °C. The extracts were then collected and centrifuged for 10 min at 16,100 × *g* at 4 °C. Finally, 200 μL of the cleared supernatant was transferred to an HPLC glass vial and stored at −80 °C until LC-MS analysis.

#### 4.6.2. LC-MS Data Acquisition

For all the experiments described above, the same analytical method was used for data acquisition. A Thermo Ultimate 3000 HPLC system coupled with a Q-Exactive Orbitrap Mass Spectrometer (Thermo Fisher Scientific, Waltham, MA, USA) was used at a resolution of 35,000 at a 200 mass/charge ratio (*m*/*z*) with electrospray ionization and a polarity switching mode to enable both positive and negative ions across a mass range of 67–1000 *m*/*z*. A spray voltage at 4.5 kV (ESI+) and 3.5 kV (ESI−), a sheath gas (nitrogen) flow rate of 25 units, an auxiliary gas (nitrogen) flow rate of 15 units, and a capillary temperature at 320 °C were all implemented. The HPLC setup consisted of ZIC-pHILIC column (at 150 mm × 2.1 mm, 5 μm; SeQuant, Merck, Darmstadt, Germany). Five microliters of biological extracts were injected, and the compounds were separated using a gradient of 15 min, which started at 20% aqueous (20 mm of ammonium carbonate adjusted to a pH of 2.0 with 0.1% of 25% ammonium hydroxide) and 80% organic (acetonitrile), and it was then terminated with 20% acetonitrile. The flow rate and column temperature were maintained at 0.2 mL/min and 45 °C, respectively, for a total run time of 27 min. All metabolites were detected using a mass accuracy below 5 p.p.m. Thermo Xcalibur version 4.1 was used for data acquisition.

#### 4.6.3. Metabolomics Data Analysis

The peak areas of the metabolites were determined using the exact mass of the singly charged ions. The mass accuracy for each identified metabolite was below 5 ppm. Its identity was further confirmed by comparing the experimental retention time (RT) to the RTs that were predetermined by analyzing an in-house mass spectrometry metabolite library of standards, which includes the IROA MSMLS (Sigma-Aldrich, St. Louis, MO, USA). TraceFinder version 4.1™ (Thermo Fisher Scientific, Waltham, MA, USA) was used for analysis. ^13^C labeling patterns were determined by integrating the peak area of the target isotopologues with the exact retention time of its unlabeled analog. The normalization of intracellular metabolites was carried out using the protein content of each extracted well that was quantified using Bradford assay. Normalization of the tissue metabolites was carried out using the tissue weight.

Some of the metabolomics methods used are described in detail in MacKay et al. [39].

### 4.7. Myotube Cell Culture

Muscle progenitor cells (MPCs) were isolated from the mice muscles, as was described in [40]. Cells were grown on collagen-coated plates in a BIO-AMF2 medium (Biological Industries, Bait Haemek, Israel). MPCs were differentiated to myotubes in a 4% horse serum-containing medium for 48 h. The myotubes were grown in the presence of glucose or palmitate as carbon sources, as described in each experiment, and the proteins were analyzed by Western blotting. For metabolomics analysis, the myotubes were supplemented with 10 mM of [U^13^]C-glucose (glucose tracing) or with 0.4 mM of palmitate-^13^C_16_ (palmitate tracing). After 6 h and 24 h incubation at 37 °C, the metabolites were extracted from replicate samples. 

### 4.8. “Seahorse” Analysis of the Oxygen Consumption Rate (OCR) and Extracellular Acidification Rate (ECAR)

Experiments were performed using an Agilent Seahorse XFe96 Extracellular Flux Analyzer (Agilent Technologies, Santa Clara, CA, USA). MPCs from the control and p38α^AF^ mice were seeded on Matrigel pre-coated XF96 plates and were incubated at 37 °C and 5% CO_2_ for 24 h. MPCs were then differentiated into myotubes in a 4% HS-containing medium for 48 h. During the last 24 h, 0.4 mM of palmitate was added to the medium. The medium was then replaced with 180 μL of unbuffered assay media (D5030 Sigma Aldrich, St. Louis, MO, USA), which was supplemented with 10 mM of glucose, 1 mM of pyruvate, and 2 mM of glutamine (with a pH of 7.4) for the mitochondrial stress test, or with 2 mM of glutamine only for the glycolysis stress test. The myotubes were then placed at 37 °C in a CO_2_-free incubator for 45 min. During the mitochondrial stress test, 2 μM of oligomycin A (Sigma Aldrich, St. Louis, MO, USA), 1.0 μM of FCCP (Sigma Aldrich, St. Louis, MO, USA), 50 μM of rotenone, and an antimycin A mixture (Sigma Aldrich, St. Louis, MO, USA) were injected sequentially. For the glycolysis stress test, the assay medium was supplemented with 2 mM of glutamine. The cells were deprived of glucose for 1 h. During the experiment, 10 mM of glucose (Sigma Aldrich, St. Louis, MO, USA), 2 μM of oligomycin A (Sigma Aldrich, St. Louis, MO, USA), and 50 mM of 2-Deoxyglucose (Sigma Aldrich, St. Louis, MO, USA) were injected sequentially. OCR and ECAR were then normalized to the protein content in each well, which was calculated at the end of the experiments using the Modified Lowry protein quantitation assay. Data were analyzed with Wave software version 2.6.

### 4.9. Detection of JC-1 Fluorescence

MPCs were cultured in a 96-well plate coated with gelatin (2 × 10^4^ cells per well). MPCs were differentiated into myotubes in a 4% HS-containing medium for 48 h. Twenty-four hours before the fluorescent analysis, the medium was changed to a differentiation medium with 0.5% BSA, or 0.4 mM of palmitate and 0.5% BSA. Mitochondrial membrane potential analysis was performed as described in [41].

### 4.10. Electron Microscopy

The muscle tissue was fixed with 2.5% glutaraldehyde and 2% paraformaldehyde in 0.1 M of sodium cacodylate buffer for 1 h at RT, which was then moved to 4°C ON. The tissue was washed with sodium cacodylate buffer, which was post-fixed and stained with 1% Osmium TetraOxide, 0.5% potassium hexacyanoferrate, and 0.5% potassium dichromate in 0.1 M of cacodylate buffer, which was followed by samples that were en-block-stained with 1% uranyl acetate for 1 h at RT. Then, the tissue was dehydrated in a graded ethanol series and embedded in Epon 812 (Electron Microscopy Sciences, Hatfield, PA, USA). Seventy nm of ultrathin sections were cut with an ultramicrotome UC7 (Leica, Wetzlar, Germany), which was then transferred to copper grids and viewed using a Zeiss Ultra-Plus FEG-SEM (Carl Zeiss Microscopy GmbH, Gottingen, Germany) equipped with a STEM detector at an accelerating voltage of 30 kV.

### 4.11. Statistical Analysis

For the mouse experiments, no specific blinding method was used, but the mice in each sample group were selected randomly. The sample size (*n*) of each experimental group is described in each corresponding figure legend, and all the experiments were repeated with at least three biological replicates. Data are expressed as the mean ± SEM, and they are represented as graphs drawn by GraphPad Prism^®^ Software V.9.0 for Windows (GraphPad Software Inc., San Diego, CA, USA) or R studio version 4.4.0. Each test (i.e., *t*-test, Wilcoxon, or ANOVA) used is detailed under the graphs. The significance levels noted were as follows: * *p* < 0.05, ** *p* < 0.01, and *** *p* < 0.001. In the metabolomics experiments, the statistical significance was described in numbers.

## 5. Conclusions

The “glucose-fatty acid cycle” proposed by Randle in 1963 described how fatty acids inhibit glucose oxidation in the mitochondria. This model has recently been reinforced by high-fat diet studies, which have demonstrated that excess lipid β-oxidation leads to metabolic inflexibility, reduced glucose oxidation, increased ROS production, and insulin resistance.

Regulating the nutrient oxidation in the mitochondria is therefore crucial for maintaining mitochondrial health. The present study’s results show that, under conditions of excessive fat calories, p38α MAPK plays a key role in regulating mitochondrial biogenesis and nutrient selection in skeletal muscle. Mitochondrial biogenesis is sustained through p38α-mediated increases in PGC1α activity, and p38α also controls the selective oxidation of fatty acids and glucose by increasing the expression of ACC2 and mediating the inhibitory phosphorylation of PDH, respectively. These dual regulatory functions of p38α prevent mitochondrial saturation, reduce respiratory burden and oxidative stress, and preserve mitochondrial integrity and insulin sensitivity.

## Data Availability

The original contributions presented in this study are included in the article/Supplementary Materials section, and further inquiries can be directed to the corresponding authors.

## Supplementary Materials

The following supporting information can be downloaded at: https://www.mdpi.com/article/10.3390/ijms25147789/s1.

## Abbreviations

MAPK	Mitogen-activated protein kinase
PDH	Pyruvate dehydrogenase
ACC2	Acetyl CoA Carboxylase 2
ND	Balanced chow diet
HFD	High-fat diet
LCFA	Long-chain fatty acid
CPT1	Carnitine palmitoyltransferase 1
Tb	Tibialis anterior
TCA	Tricarboxylic acid
AMPK	AMP-activated protein kinase
PGC-1α	Peroxisome proliferator-activated receptor gamma coactivator 1-alpha
ITT	Insulin tolerance test
ROS	Reactive oxygen species
