# Supplementary material for: P38α MAPK Coordinates Mitochondrial Adaptation to Caloric Surplus in Skeletal Muscle
Source: Int J Mol Sci. 2024 Jul 16;25(14):7789. doi: 10.3390/ijms25147789 (PMC11277080; doi:10.3390/ijms25147789)
Supplement: Supplementary file 1 [file ijms-25-07789-s001.zip › Supplementary Figures and Supplementary Materials/Supplementary figures IJMS 15.7.24.pptx]

## Slide 1
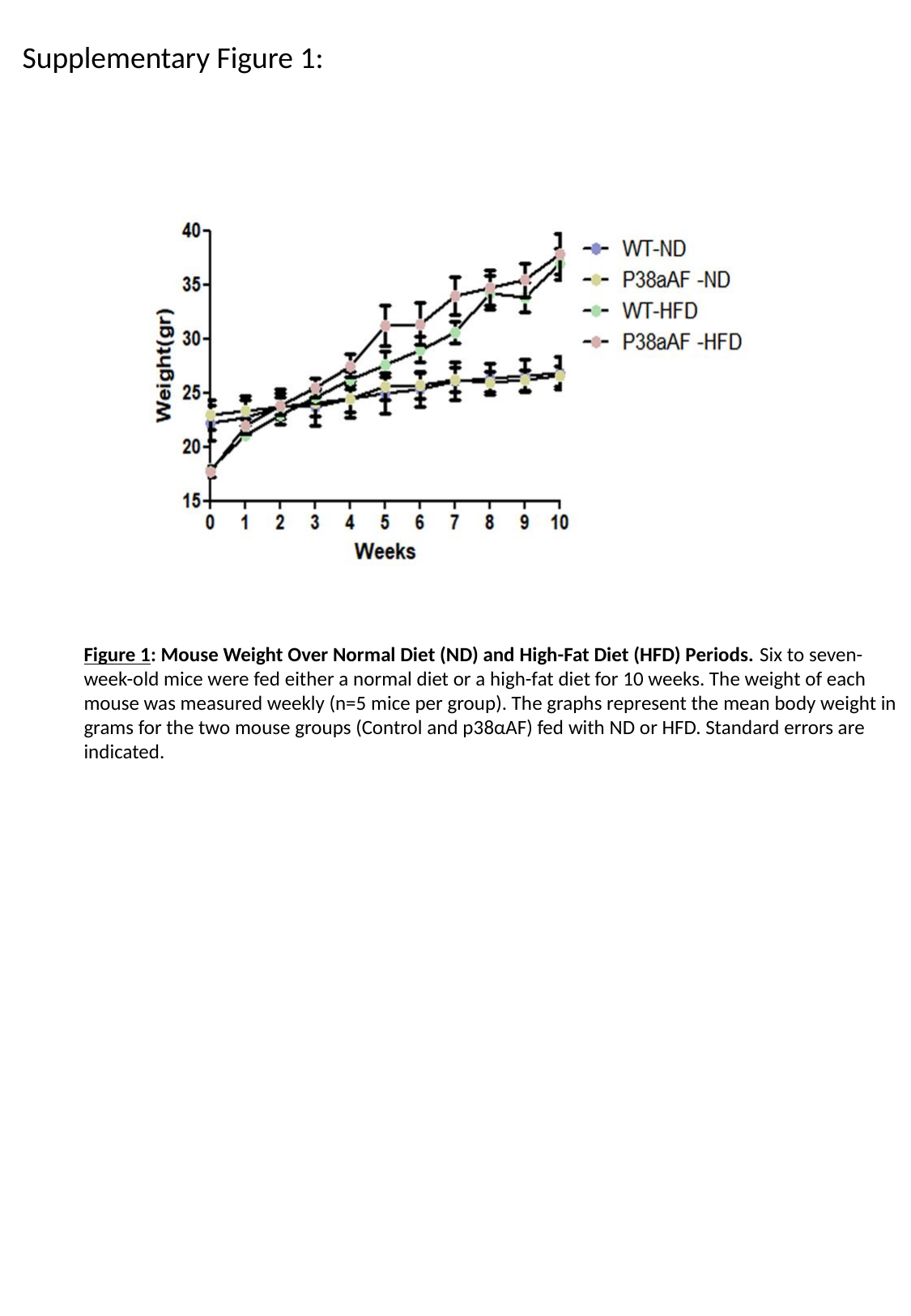

Supplementary Figure 1:
Figure 1: Mouse Weight Over Normal Diet (ND) and High-Fat Diet (HFD) Periods. Six to seven-week-old mice were fed either a normal diet or a high-fat diet for 10 weeks. The weight of each mouse was measured weekly (n=5 mice per group). The graphs represent the mean body weight in grams for the two mouse groups (Control and p38αAF) fed with ND or HFD. Standard errors are indicated.

## Slide 2
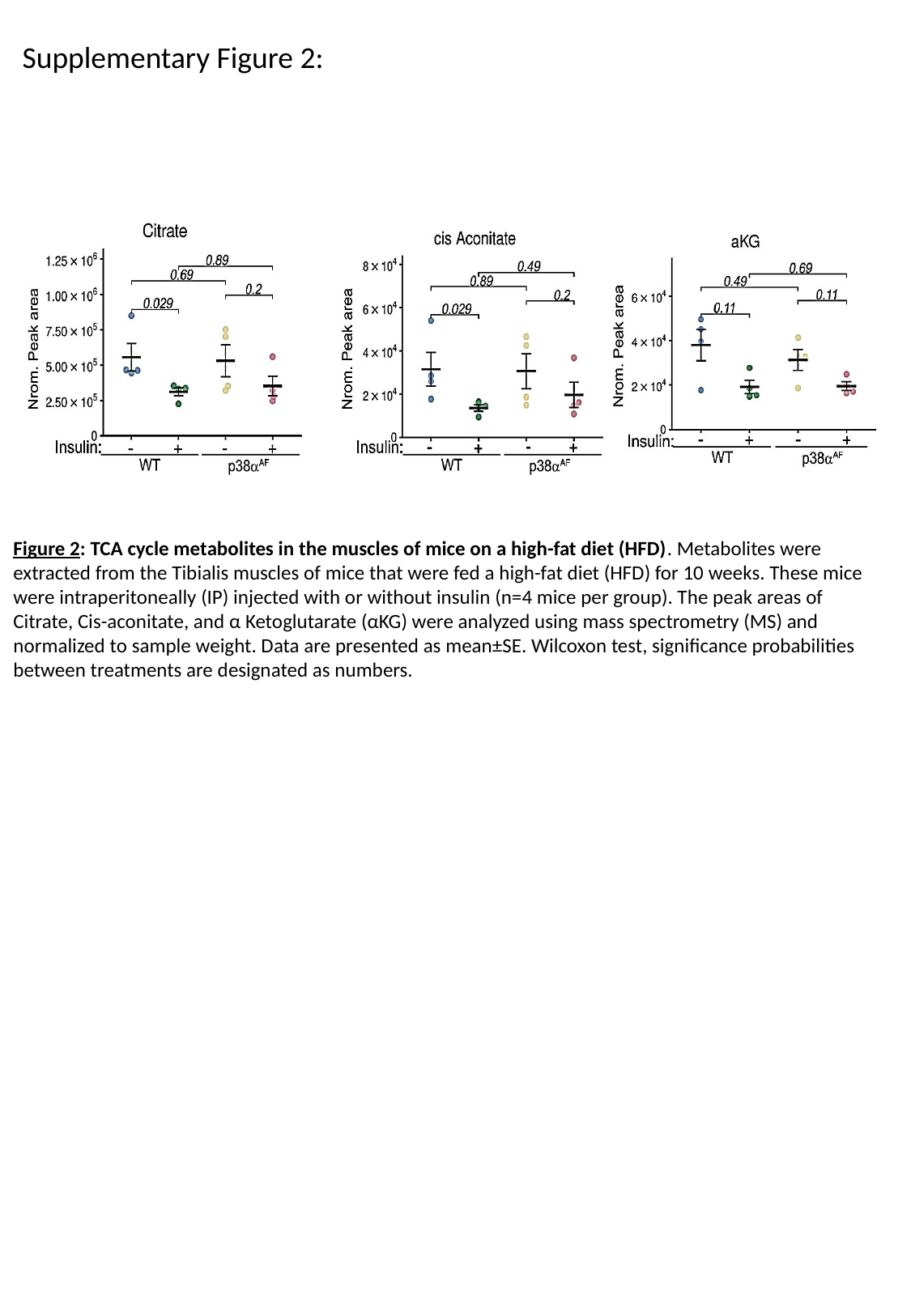

Supplementary Figure 2:
Figure 2: TCA cycle metabolites in the muscles of mice on a high-fat diet (HFD). Metabolites were extracted from the Tibialis muscles of mice that were fed a high-fat diet (HFD) for 10 weeks. These mice were intraperitoneally (IP) injected with or without insulin (n=4 mice per group). The peak areas of Citrate, Cis-aconitate, and α Ketoglutarate (αKG) were analyzed using mass spectrometry (MS) and normalized to sample weight. Data are presented as mean±SE. Wilcoxon test, significance probabilities between treatments are designated as numbers.

## Slide 3
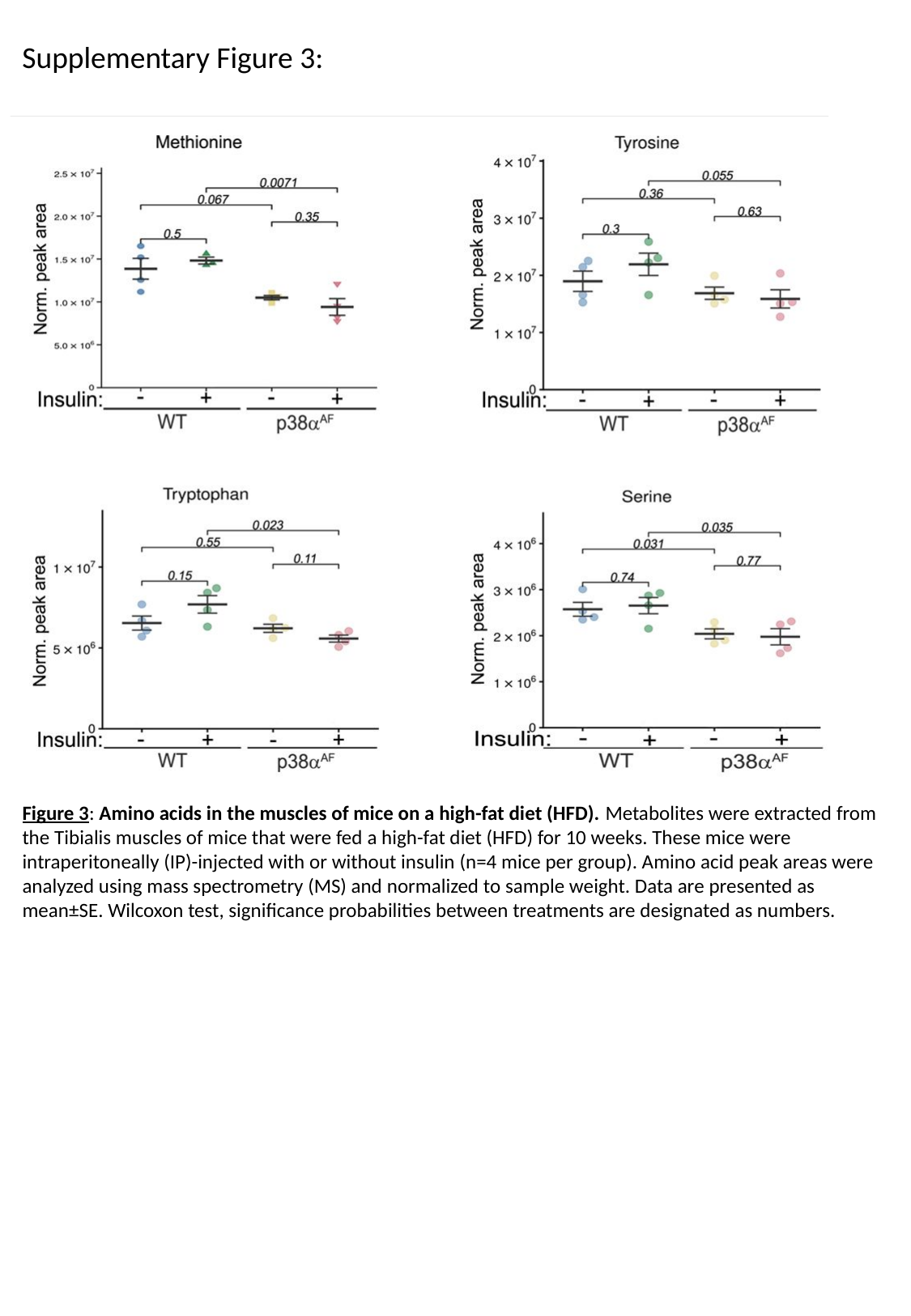

Supplementary Figure 3:
Figure 3: Amino acids in the muscles of mice on a high-fat diet (HFD). Metabolites were extracted from the Tibialis muscles of mice that were fed a high-fat diet (HFD) for 10 weeks. These mice were intraperitoneally (IP)-injected with or without insulin (n=4 mice per group). Amino acid peak areas were analyzed using mass spectrometry (MS) and normalized to sample weight. Data are presented as mean±SE. Wilcoxon test, significance probabilities between treatments are designated as numbers.

## Slide 4
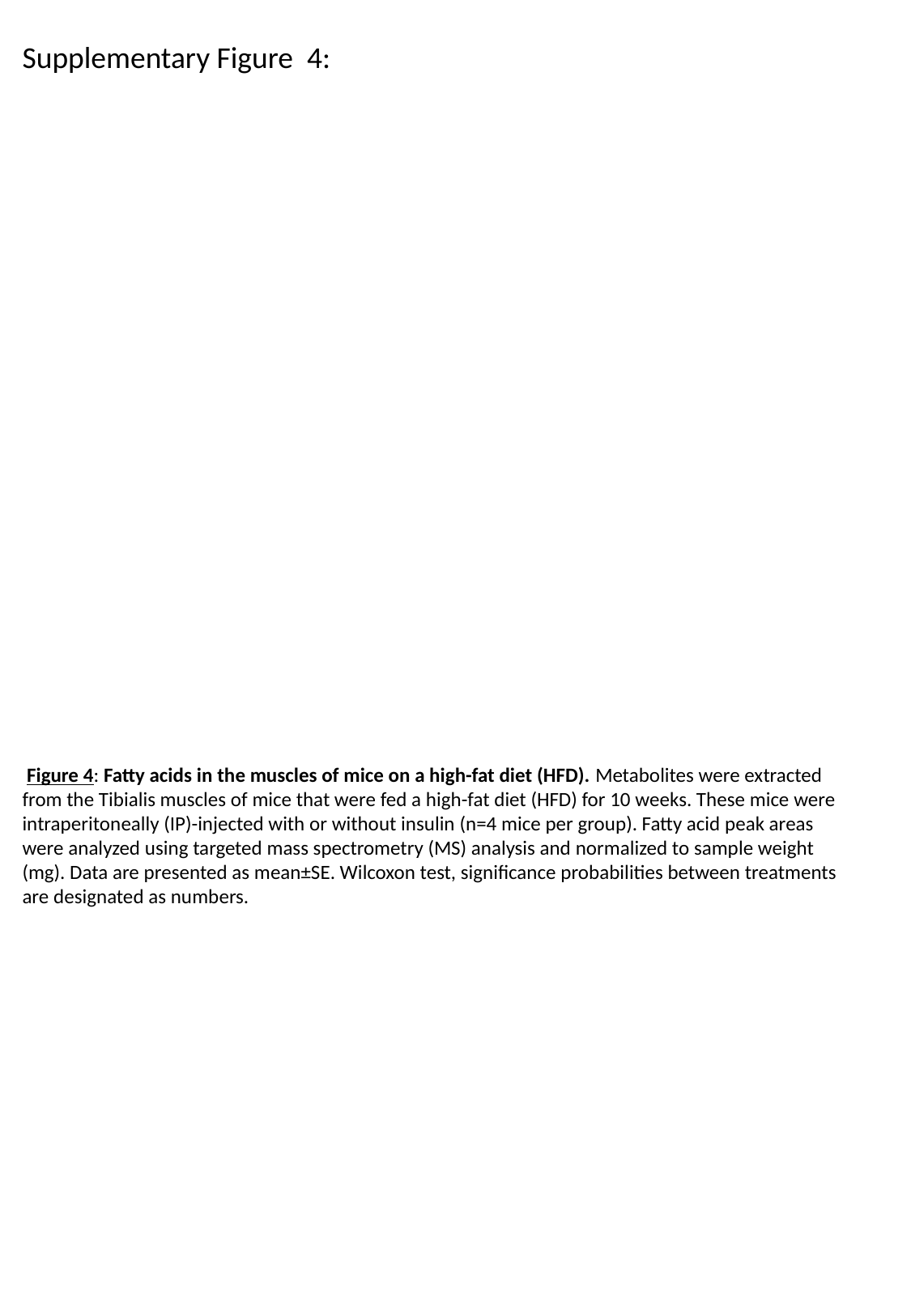

Supplementary Figure 4:
 Figure 4: Fatty acids in the muscles of mice on a high-fat diet (HFD). Metabolites were extracted from the Tibialis muscles of mice that were fed a high-fat diet (HFD) for 10 weeks. These mice were intraperitoneally (IP)-injected with or without insulin (n=4 mice per group). Fatty acid peak areas were analyzed using targeted mass spectrometry (MS) analysis and normalized to sample weight (mg). Data are presented as mean±SE. Wilcoxon test, significance probabilities between treatments are designated as numbers.

## Slide 5
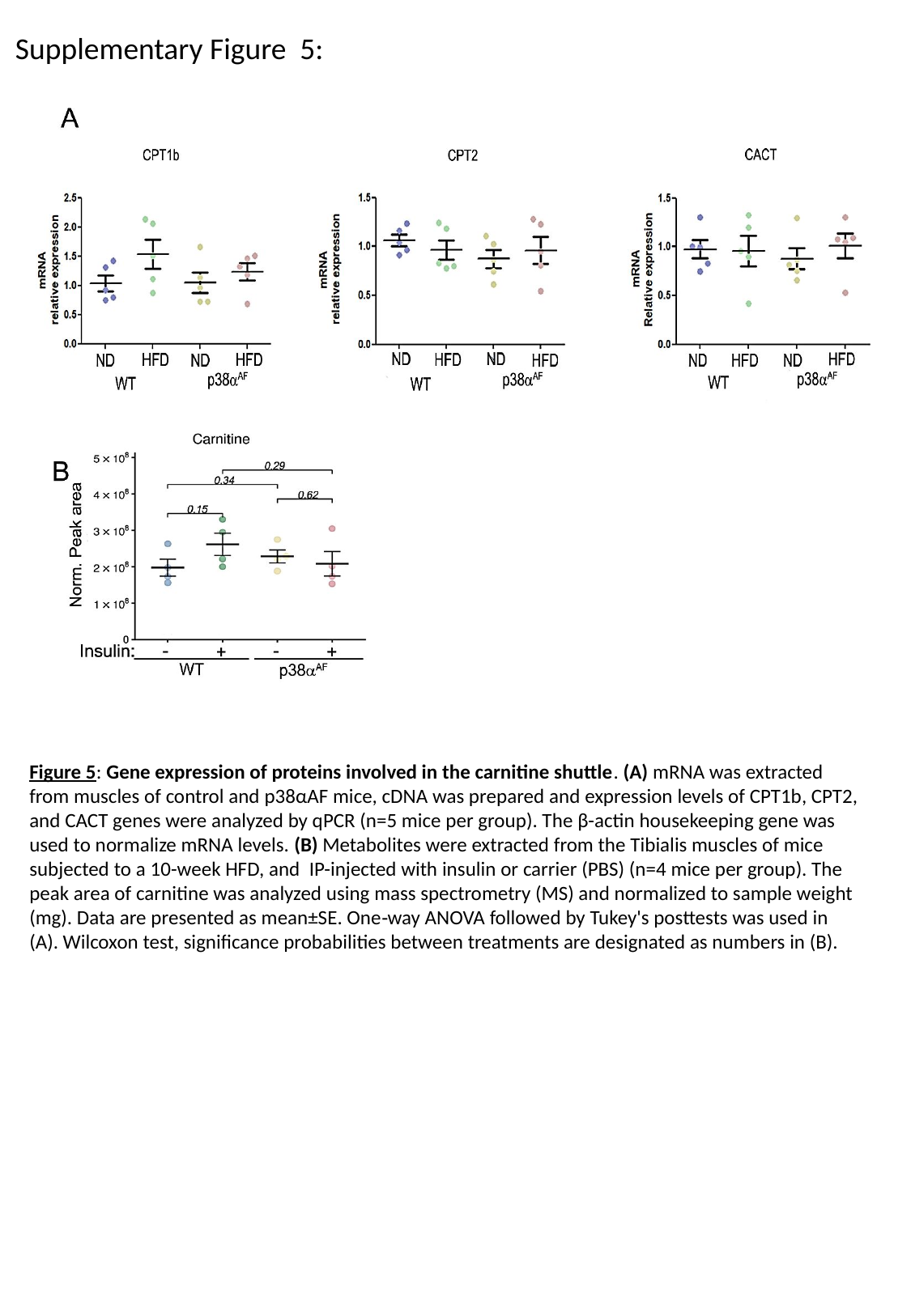

Supplementary Figure 5:
Figure 5: Gene expression of proteins involved in the carnitine shuttle. (A) mRNA was extracted from muscles of control and p38αAF mice, cDNA was prepared and expression levels of CPT1b, CPT2, and CACT genes were analyzed by qPCR (n=5 mice per group). The β-actin housekeeping gene was used to normalize mRNA levels. (B) Metabolites were extracted from the Tibialis muscles of mice subjected to a 10-week HFD, and IP-injected with insulin or carrier (PBS) (n=4 mice per group). The peak area of carnitine was analyzed using mass spectrometry (MS) and normalized to sample weight (mg). Data are presented as mean±SE. One‐way ANOVA followed by Tukey's posttests was used in (A). Wilcoxon test, significance probabilities between treatments are designated as numbers in (B).

## Slide 6
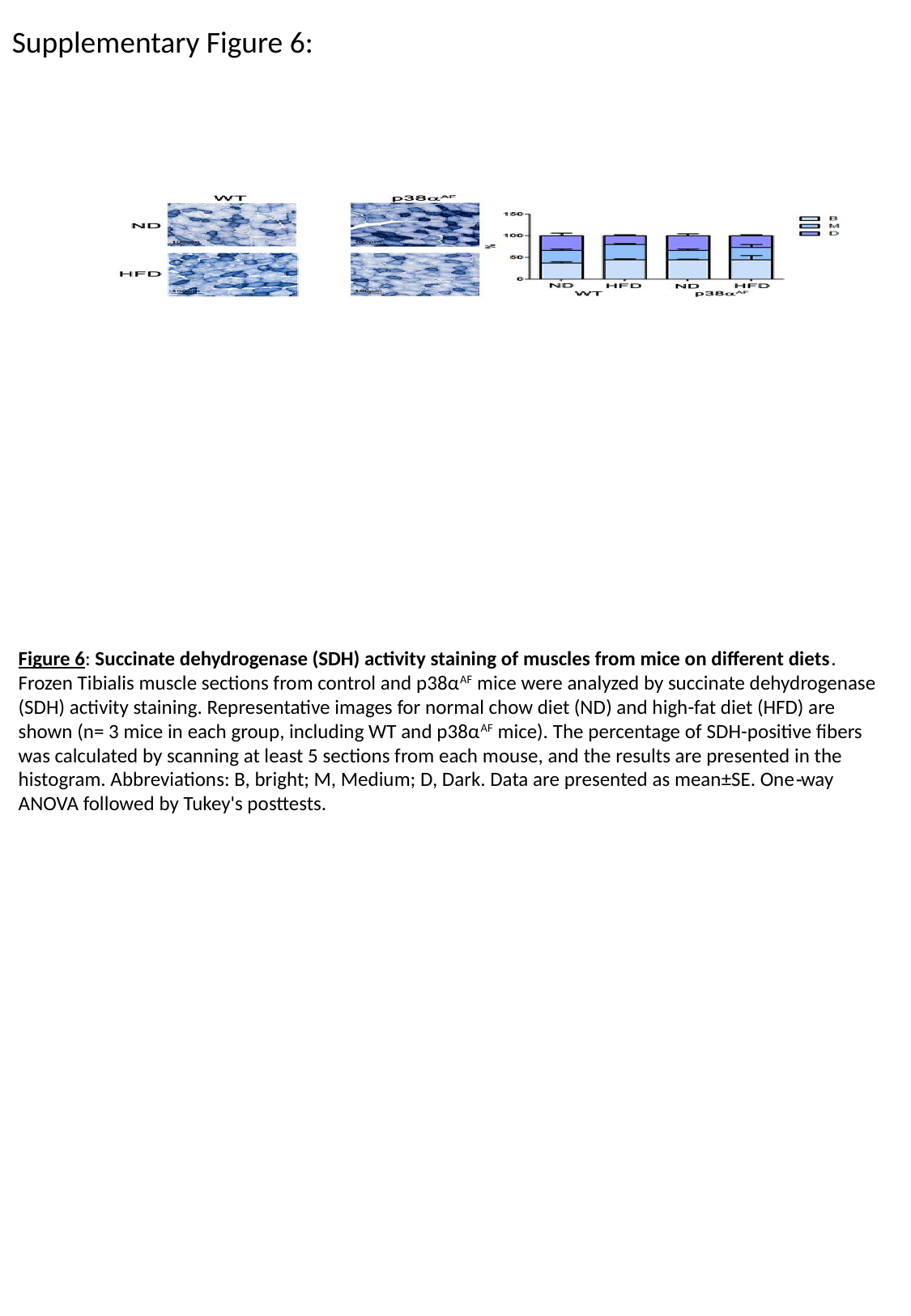

Supplementary Figure 6:
Figure 6: Succinate dehydrogenase (SDH) activity staining of muscles from mice on different diets. Frozen Tibialis muscle sections from control and p38αAF mice were analyzed by succinate dehydrogenase (SDH) activity staining. Representative images for normal chow diet (ND) and high-fat diet (HFD) are shown (n= 3 mice in each group, including WT and p38αAF mice). The percentage of SDH-positive fibers was calculated by scanning at least 5 sections from each mouse, and the results are presented in the histogram. Abbreviations: B, bright; M, Medium; D, Dark. Data are presented as mean±SE. One‐way ANOVA followed by Tukey's posttests.
