# Supplementary material for: P38α MAPK Coordinates Mitochondrial Adaptation to Caloric Surplus in Skeletal Muscle
Source: Int J Mol Sci. 2024 Jul 16;25(14):7789. doi: 10.3390/ijms25147789 (PMC11277080; doi:10.3390/ijms25147789)
Supplement: Supplementary file 1 [file ijms-25-07789-s001.zip › Supplementary Figures and Supplementary Materials/Supplementary Materials 15.7.24.docx]

**Supplementary Materials:**

**1. Antibodies used in Western analysis:**

Primary antibodies used in this study:

• Anti Phospho-p38 MAP Kinase (Thr180/Tyr182) (Cell Signaling, #9211) diluted 1:500.

• Anti αTubulin (sigma, T9026) diluted 1:10000.

• Anti Phospho AMPKα (Thr172) (Cell Signaling, #2531) diluted 1:1000.

• Anti AMPKα (Cell Signaling, #2532) diluted 1:1000 for western blot.

• Anti HSP27 (Cell Signaling, #2442) diluted 1:1000.

• Anti Phospho - p70 S6 Kinase (Thr389) (108D2) (Cell Signaling, #9234) diluted 1:1000.

• Anti p70 S6 Kinase (49D7) (Cell Signaling, #2708) diluted 1:1000.

• Anti p38α MAP Kinase (Cell Signaling, #9218) diluted 1:1000.

• Anti p38γ MAPK (Cell Signaling, #2307) diluted 1:1000

• Anti Akt (pan) (C67E7) (Cell signaling Rabbit mAb #4691) diluted 1:1000

• Anti Phospho-Akt (Ser473) (Cell Signaling (D9E) XP^®^ Rabbit mAb #4060) diluted 1:1000

• Anti Phospho-Acetyl-CoA Carboxylase (Ser79) (D7D11) (Cell Signaling, #11818) diluted 1:1000.

• Anti Acetyl-CoA Carboxylase 2 (D5B9) (Cell Signaling, #8578) diluted 1:1000.

• Anti Citrate synthase (D7V8B) (Cell Signaling, #14309) diluted 1:1000.

• Anti PDH (Cell Signaling, (C54G1) Rabbit mAb #3205) diluted 1:1000

• Anti Phospho-Pyruvate Dehydrogenase α1(Ser293) (Cell Signaling, (E4V9L) Rabbit mAb #37115) 1:1000

• Anti GSK-3α/β (Cell Signaling, (D75D3) Rabbit mAb #5676) Diluted 1:1000

• Anti Phospho-GSK-3α/β (Ser21/9) (Cell Signaling, (D17D2) Rabbit mAb #8566) diluted 1:1000

**2. List of Primers used in real-time PCR analysis:**

| **Primer name** | **seq** |  |
| --- | --- | --- |
| FABP3 Forward | CCCCTCAGCTCAGCACCAT |  |
| FABP3 Reverse | CAGAAAAATCCCAACCCAAGAAT |  |
| PGC1α Forward | TATGGAGTGACATAGAGTGTGCT |  |
| PGC1α Reverse | CCACTTCAATCCACCCAGAAAG |  |
| CPT1b Forward | GCACACCAGGCAGTAGCTTT |  |
| CPT1b Reverse | CAGGAGTTGATTCCAGACAGGTA |  |
| CPT2 Forward | CAGCACAGCATCGTACCCA |  |
| CPT2 Reverse | TCCCAATGCCGTTCTCAAAAT |  |
| CACT Forward | GACGAGCCGAAACCCATCAG |  |
| CACT Reverse | AGTCGGACCTTGACCGTGT |  |
| ACC2 Forward | CGCTCACCAACAGTAAGGTGG |  |
| ACC2 Reverse | GCTTGGCAGGGAGTTCCTC |  |
| β-actin Forward | GCTCTGGCTCCTAGCACCAT |  |
| β-actin Reverse | CCACCGATCCACACAGAGTAC |  |
